# Ferroptosis-Resistant Adipocytes Drive Keloid Pathogenesis via GPX4-Mediated Adipocyte-Mesenchymal Transition and Iron-Cystine Metabolic Communication

**DOI:** 10.7150/ijbs.114930

**Published:** 2025-07-28

**Authors:** Xiangguang Shi, Xueyi Xia, Yang Xiao, Huizhen Shu, Zhuoya Xu, Mengguo Liu, Chenyi Shi, Ying Zhang, Yining Wei, Yiyi Gong, Wei Wang, Yahui Chen, Jianlan Liu, Jia Huang, Mengkun Shi, Jiucun Wang, Wenyu Wu

**Affiliations:** 1 Department of Dermatology, Huashan Hospital, Deptartment of Allergy and immunology, Huashan Hospital, and Research Center of Allergy and Diseases, Shanghai Institute of Dermatology, State Key Laboratory of Molecular Engineering of Polymers, Fudan University, Shanghai, China.; 2 State Key Laboratory of Genetic Engineering, Collaborative Innovation Center for Genetics and Development, School of life science and Human Phenome Institute, Fudan University, Shanghai, China.; 3 Department of Thoracic Surgery, Huashan Hospital & Cancer Metastasis Institute, Fudan University, Shanghai, China.; 4 Institute of Rheumatology, Immunology and Allergy, Fudan University, Shanghai, China.; 5 Research Unit of Dissecting the Population Genetics and Developing New Technologies for Treatment and Prevention of Skin Phenotypes and Dermatological Diseases (2019RU058), Chinese Academy of Medical Sciences, Shanghai, China.; 6 Department of Dermatology, Jing'an District Central Hospital, Shanghai, China.; 7 National Clinical Research Center for Aging and Medicine, Huashan Hospital, Fudan University, Shanghai, China.; 8 Ministry of Education Key Laboratory of Contemporary Anthropology, School of Life Sciences, and Academy for Engineering and Technology, Fudan University, Shanghai, China.

**Keywords:** Keloid, GPX4, Adipocyte-Mesenchymal Transition, ferroptosis resistance, iron overload, interferon suppression.

## Abstract

**Background:** Keloids are a challenging fibrotic disorder with limited treatment options. The study sought to examine the underlying mechanisms of keloid pathogenesis, emphasizing the influence of dermal adipocytes and ferroptosis resistance in driving fibrosis.

**Methods:** Single-cell RNA sequencing (scRNA-seq) was employed for determining essential cell populations in keloid tissue. Mechanistic studies assessed iron overload, Reactive Oxygen Species (ROS) exhaustion, and interferon responses in ferroptosis-resistant adipocytes. Glutathione peroxidase 4 (GPX4) expression and TGF-β signaling activation were evaluated in adipocyte-mesenchymal transition (AMT). Paracrine signaling and metabolic symbiosis between adipocytes and fibroblasts were analyzed. Therapeutic interventions (ferroptosis inducer RSL3 and iron chelator deferoxamine DFO) were tested *in vivo*.

**Results:** Through single-cell RNA sequencing, we identified ferroptosis-resistant dermal adipocytes as key contributors to keloid pathogenesis, exhibiting iron overload, ROS suppression, and impaired interferon responses. These adipocytes demonstrated elevated GPX4 expression, which mechanistically drove AMT via iron-dependent activation of TGF-β signaling pathways. GPX4-activated adipocytes promoted fibroblast collagen production through paracrine signaling while establishing a metabolic symbiosis: adipocytes exported iron via solute carrier family 40 member 1 (SLC40A1) to neighboring fibroblasts, which reciprocally supplied cystine through cystathionine beta-synthase (CBS)/cystinosin, lysosomal cystine transporter (CTNS) to sustain GPX4 activity. This vicious cycle was further amplified by iron/ROS-mediated suppression of interferon signaling, creating a pro-fibrotic feedback loop. Therapeutic targeting with either the ferroptosis inducer RSL3 or iron chelator deferoxamine (DFO) effectively disrupted this pathological network, suppressing GPX4/AMT while restoring interferon responses and attenuating keloid growth *in vivo*. This study clarifies a new adipocyte-focused mechanism in keloid development and identifies ferroptosis regulation as a potential treatment approach for this persistent condition.

Conclusions: This study reveals a novel adipocyte-centered mechanism in keloid pathogenesis driven by GPX4-mediated ferroptosis resistance, metabolic symbiosis, and disrupted interferon signaling**.** The findings establish ferroptosis modulation (via RSL3 or iron chelation) as a promising therapeutic strategy for keloids, offering potential new treatments for this recalcitrant condition.

## Introduction

Keloids typically develop subsequent to skin injury 1. They are characterized by being larger and more prominent compared to the original wounds. The pathogenesis of keloid remains unknown, and effective therapeutic targets are lacking. Although some drugs, therapy, laser therapy, surgery, and other treatments have demonstrated a certain effect on keloid, the scar cannot be completely eradicated, and the recurrence rate is high 2, 3.

Keloid formation is associated with various biological mechanisms, including abnormal proliferation of fibroblasts and inflammatory responses 3. In recent years, alterations in the adipose tissue of subcutaneous fat have drawn increasing attention in skin diseases. Adipose tissue serves as a reservoir for energy storage and participates in numerous biological processes, including the regulation of metabolism, endocrine activity, and immunological responses 4. Adipose consists of adipocytes, stromal cells, and immune cells, thus playing a complex role in cellular interactions. Keloids often exhibit hardening and thickening characteristics, which may also impact the structure of the surrounding adipose tissue. Hardened fibrotic tissue might also exert pressure on surrounding adipocyte cells, causing them to function abnormally, such as in scleroderma, where a considerable amount of fat tissue is lost 5-8. In addition, adipocyte cells can store fat and secrete various bioactive substances, such as adipokines. During skin fibrosis, the function of adipocyte cells might be affected, resulting in abnormal secretion of these factors, which influences local healing processes, inflammation, and repair responses 5. In addition, the apoptosis of fibrotic skin fibroblasts is inhibited, leading to their excessive proliferation, and these processes may also be closely related to adipose tissue changes 9. Furthermore, it has been reported that adipose tissue can be transformed into fibroblasts through Adipocyte-Mesenchymal Transition (AMT) to exacerbate SSc^7^. AMT contributes to SSc by converting subcutaneous adipocytes into fibroblast-like cells under pathological conditions. This process increases the pool of ECM-producing myofibroblasts, exacerbating collagen deposition and tissue stiffness. Principal regulators encompass the TGF-β, Wnt/β-catenin, and PPAR-γ pathways, with hypoxia and oxidative stress further promoting AMT. AMT-derived cells exhibit enhanced migratory and fibrogenic properties, driving fibrosis progression. Targeting AMT may offer therapeutic potential in fibrotic skin disorders. However, whether adipocyte abnormalities exist in KD, how adipocytes interact with fibroblasts, and whether they are involved in KD and its mechanisms remain unknown.

Iron is a vital element for cellular metabolism and is involved in numerous physiological activities, including oxygen transport and energy synthesis 10. However, excessive iron can generate free radicals, which can trigger cell damage and death. It has been reported that in obese patients, the intake of iron increases, leading to the occurrence of iron accumulation in adipocyte cells, which impacts their function and viability 11. Ferroptosis is a unique form of cell death characterized by iron dependence, accumulation of lipid peroxides, and reduction of glutathione peroxidase 4 (GPX4) 12. Recent discoveries demonstrate a substantial correlation between iron overload and the functionality of adipocyte cells, particularly in the regulation of energy metabolism and cell death 13. Although iron overload can cause ferroptosis by the generation of reactive oxygen species (ROS) via the Fenton reaction, in some diseases, such as obesity, cells in adipose tissue protect against ferroptosis by upregulating GPX4 to enhance antioxidant mechanisms. In many malignancies, cancer cells can also resist cell ferroptosis caused by ROS and others through high expression of GPX4^14^, ^15^. Therefore, an in-depth investigation of the function and mechanism of iron metabolism abnormality and ferroptosis in adipocytes is anticipated to develop new strategies for treating KD.

Keloids are often accompanied by a vigorous inflammatory response. Interferons can be categorized into three types: alpha, beta, and gamma 16. Interferon can enhance collagenase activity, inhibit collagen synthesis, regulate immunity, and reduce the inflammatory response, which might have an inhibitory effect on KD 17. Studies have shown that adipocyte cells can secrete several types of interferons, especially interferon-gamma (IFN-γ) and interferon-alpha (IFN-α) 18. Research has shown that two local injections of interferon alpha-2B can diminish the size of keloids. However, this remains a subject of debate 19. Interferon (IFN) also possesses antiviral and antibacterial activities. In addition, interferon is also utilized in some types of cancer treatment 20. It has been reported that keloids can be associated with certain bacterial infections. Bacterial diseases, such as leprosy, may lead to the formation of keloid 21. In addition, bacterial infections may increase the incidence of keloids, especially in the presence of postoperative infection 22. It can be perceived that the regulation of interferon in adipocytes will have beneficial effects on KD collagen synthesis, inflammatory immunity, and bacteria and viruses.

In this study, we initially discovered that there was iron overload, ferroptosis resistance, and inhibition of interferon signaling in KD adipocytes through single-cell research. After the overexpression of GPX4 in adipocytes, the above phenomenon can be replicated, and AMT can be significantly induced to produce a large amount of collagen. Regarding the mechanism, GPX4-overexpressed adipocytes acquire ferroptosis resistance and, on the one hand, induce AMT in adipocytes through iron overload, and on the other hand, deliver iron ions to fibroblasts through iron export protein SLC40A1 to promote fibroblast proliferation and fibrosis reaction. Ferroptosis resists excessive depletion of ROS within adipocyte cells and iron overload, which in turn leads to interferon non-response, enhancing the inflammatory response. Fibroblasts maintain high GPX4 activity by synthesizing and secreting cystine, which is absorbed by system Xc^-^ on the surface of adipocyte cells.* In vivo* mouse experiments, the inhibition of GPX4 by using RSL3 and blockage of GPX4-induced iron overload by DFO can restore the sensitivity of adipocyte cells to ferroptosis and effectively inhibit KD. Collectively, we elucidated the pivotal function of adipocytes in keloids. The management of KD will improve by suppressing abnormal adipocyte-fibroblast communication and obstructing iron overload, ferroptosis resistance, and impairment of interferon signaling.

## Materials and methods

### Patients

According to the JSW Scar Scale (JSS) 2015 categorization and evaluation criteria, keloid patients at Shanghai Huashan Hospital provided the skin samples used in this investigation 23. Normal skin tissue (KN) adjacent to keloid (KD) tissue from the same patient, taken concurrently during the surgical operation, is referred to as paired keloids. The tissues KN and KD are classified as paired tissues. Unpaired tissues are samples taken from various people that only contain either normal or KD skin tissue after surgery. The appropriately region-matched keloid provided the control unpaired KN samples. All participants gave their informed consent after receiving a comprehensive briefing on the study prior to its start. The study was approved by Fudan University's ethics board (Approval no. KY2023-015);** Supplementary Table S2** provides more details on the patients and normal controls.

### Perl's staining and iron assay

The manufacturer's instructions for Perl's staining (Hematognost Fe®, 112,084, Sigma-Aldrich, St. Louis, MO, USA) were followed. A counterstain of 0.1% nuclear fast red was used after the slices had been treated with 5% potassium hexacyanoferrate and 5% hydrochloric acid. Slices were cleaned in distilled water, dehydrated using a sequence of increasing alcohol concentrations, and then covered with a cover slip before being photographed using light microscopy (NIKON ECLIPSE C1). Using an iron assay kit from Dojindo (catalog no. I291, Iron Assay Kit- Colorimetric), the amounts of total iron, Fe^2+^, and Fe^3+^ in cultured cells or skin tissues were measured in accordance with the manufacturer's instructions. Thermo Fisher Scientific, Inc.'s Multiskan GO Microplate Spectrophotometer was used to measure each sample's absorbance at 593 nm.

### Cell cultures

As previously described, cutaneous primary fibroblasts were isolated from both normal controls and keloid patients 20. Following an initial disinfection in 75% ethanol, skin biopsy specimens were washed three times in phosphate-buffered saline (PBS) treated with penicillin and streptomycin. After being broken up, the corium layer specimens were placed on culture dishes. After two hours of reverse culture, DMEM was added to the cell culture medium. For further analysis, primary cutaneous fibroblasts from the third and fifth passages were used.

### Isolation of primary adipocyte cells

After chopping the adipose tissue finely with scissors, it was digested for 45 minutes at 37°C using 1.5 mg/ml collagenase type II (Sigma, Munich, DE) in DMEM and 0.5% fatty acid-free bovine serum albumin (BSA) (Sigma, Munich, DE) and 15 mM HEPES. Following a 10-minute rest period, the floating mature adipocyte fraction was extracted from the cell suspensions, washed with full media, and filtered through a 300 µm mesh. After that, the cells were cultured in DMEM with 10% FCS and 1% penicillin/streptomycin added.

### Creation of the human keloid-bearing murine model

The procedures followed in the use of Balb/c nude mice for this work were approved by Fudan University's Institutional Animal Care and Use Committee (Approval number: FE20002). Keloid biopsy specimens were sectioned into 5 mm cubes from the dermis and subcutaneous white adipose tissue, maintaining equivalent weights. Prior to inserting one cube into a 1-centimeter incision just below the right shoulder blade for the implantation of keloid tissues, six to eight-week-old naked mice were anesthetized with 1.25% tribromoethanol. After that, the wound was sutured. Following a 14-day period of separate housing under standard conditions, the keloid-bearing mice were prepared for the subsequent phase of testing. Keloid-bearing mice underwent injections of 50 mg/kg RSL3 or 100 mg/kg DFO every three days for a total of eight administrations prior to harvesting.

### Histological and immunofluorescence detection

The keloid and nearby normal tissue samples were first fixed in 10% paraformaldehyde before being embedded in paraffin for immunofluorescence (IF), Masson's staining, Masson-Fontana staining, and hematoxylin and eosin (H&E) staining. The standard operating procedures for H&E, Masson's staining, and Masson-Fontana staining were followed. Anti-S100A4 (1:100), anti-PLIN1 (1:500), anti-GPX4 (1:100), anti-FTH1 (1:100), anti-OAS1/3 (1:500), anti-p-IRF3 (1:400), anti-CBS (1:500), anti-CTNS (1:500), and anti-α-SMA (1:100) were the main antibodies used to incubate the samples in order to perform IHC and IF staining. The samples were then treated with secondary antibodies that were either Cy3-conjugated Anti-Rabbit (1:100) or FITC-conjugated Anti-Mouse (1:100). The IHC slices were seen using DAB peroxidase substrate, while hematoxylin was used as a counterstain. The nuclei and immunofluorescence sections were counterstained with DAPI. Fluorescence confocal pictures were taken using a NIKON ECLIPSE C1.

### RNA extraction, cDNA synthesis, and qRT-PCR

An RNA Isolation Kit was used to isolate total RNA from keloid and normal control primary fibroblasts and skin tissues from the third to fifth passages. Reverse transcriptase synthesized cDNA with HiScript II 1st Strand cDNA Synthesis Kit. The Roche-LC480 Real-Time PCR equipment (Roche, Switzerland) was used for SYBR-Green real-time qRT-PCR.** Supplementary Table S3** lists primers. The expression of each gene was compared to β-actin or GAPDH.

### Protein extraction and Western blotting

The manufacturer's instructions were followed to extract protein from primary fibroblasts and skin tissues of keloid and normal controls. After fractionation with 10% SDS-PAGE, total proteins were deposited onto Millipore 0.45 µm nitrocellulose membranes. Primary antibodies were incubated overnight at 4°C after blocking with 5% BSA/TBST. Next, HRP-conjugated secondary antibodies were added. The primary antibodies utilized in western blot analysis included anti-collagen type I (A22090, ABclonal), anti-α-SMA (GB111364, Servicebio), anti-Phospho-SMAD2/3 (8828, CST), anti-Phospho-Akt (4060, CST), anti-Phospho-p44/42 MAPK (4370, CST), anti-Phospho-p38 MAPK (4511, CST), anti-FTH1 (4393, CST), anti-GPX4 (67763-1-Ig, Proteintech), anti-SLC3A2 (47213, Proteintech), anti-SLC7A11 (26864-1-AP, Proteintech), anti-SLC40A1 (NBP1-21502, NOVUS), anti-ZIP8 (20459-1-AP, Proteintech), anti-TFRC (ab214039, Abcam), anti-ACSL4 (ab155282, Abcam), anti-MX1 (ab284603, Abcam), anti-Perilipin1 (27716-1-AP, Proteintech), anti-IFNAR2 polyclonal (10522-1-AP, Proteintech), anti-Phospho-IRF-3 (29047, CST), anti-CBS (14782, CST), anti-CTNS (13085-1-AP, Proteintech), and anti-OAS1/3 polyclonal (14955-1-AP, Proteintech). Each experiment was conducted independently three times. The expression levels of each protein were compared to β-actin or GAPDH.

### Cell viability and proliferation assessment

Using CCK-8, cell viability was assessed. In 96-well plates, fibroblast or melanocyte cells (1×10^4 cells/well) were cultured for a whole day. RSL3 was added and cultivated for an additional 24 hours after the culture medium was removed. Ten microliters of CCK-8 solution were added to each well before the CCK8 assay. A microplate spectrophotometer was used to detect absorbance at 450 nm after two hours of incubation. After washing the cells with PBS, trypsin digested them, and culture media stopped the process. The Countstar Mira FL (Alit Biotech (Shanghai) Co., Ltd.) was used to measure the rate of cell growth.

### Assessment of cystine absorption

Cystine uptake was measured using the Cystine uptake assay kit (No. UP05, Dojindo Laboratories) in accordance with the manufacturer's instructions. The Tecan microplate reader was used to measure the fluorescence. The adjacent wells were shielded from light leakage by a black 96-well panel.

### Migration and Invasion Assay

In order to perform the scratch wound test, cells were seeded onto 12-well plates. After the cells reached 100% confluence, they were washed with PBS and scraped with a 200 µl pipette tip to wound them. The cells were cultivated in an adipocyte medium, either with or without GPX4-OE pretreatment, after three PBS washes. We took pictures of the wound site at 0, 24, 48, and 72 hours.

### MDA and Lipid Reactive Oxygen Species (LipidROS) Assay

A lipid peroxidation test kit (M496, Dojindo Laboratories, Kumamoto, Japan) was used to measure the levels of MDA. As directed by the manufacturer, Liperfluo (L248, Dojindo) was used to stain lipid peroxide. After adding liperfluo to the medium, fibroblast cells were cultivated for an hour at 37°C. Using a fluorescent microscope (NIKON ECLIPSE C1), fibroblasts were investigated.

### Sircol assay

The collagen protein concentration in the cell media was adjusted to the total protein content, and the total soluble collagen content in the medium was measured using the Sircol assay kit in accordance with the manufacturer's instructions.

### RNA interference

HANBIO produced the *SLC40A1* siRNA sequences: Forward 5ʹ-GGACAAUCACAACCUGAUUTT-3ʹ and Reverse 5ʹ-AAUCAGGUUGUGAUUGUCCTT-3ʹ.

The non-targeting control siRNA (*NC*) consisted of scrambled sequences: Forward 5'-UUCUCCGAACGUGUCACGUTT-3' and Reverse 5'-ACGUGACACGUUCGGAGAATT-3'. LipofectamineTM RNAiMAX Transfection Reagent transfected 30 picomoles of siRNA or negative control. After 48 hours of transfection, cells were collected for examination.

### RNA sequencing (RNA-seq)

Following the manufacturer's instructions, fibroblast cells were used to extract total RNA and create a cDNA library using a KAPA RNA HyperPrep kit (Kapa Biosystems, Wilmington, MA, USA). The cDNA libraries were sequenced using the Illumina HiSeq X Ten system (Illumina, USA). The transcriptome data was analyzed by Deseq2 and Kallisto. Genes having a significance level of P < 0.05 are categorized as differentially expressed genes (DEGs).

### Single-cell RNA sequencing (scRNA-seq)

Six neighboring skin donors (KN) and six keloid sufferers (KD) had their skin samples chopped, enzymatically dissociated, and cleaned with PBS. To create single-cell suspensions, the epidermal and dermal layers were separated, then treated with trypsin or collagenase P/Dnase I and filtered. After digestion, single-cell reagent kits (3' v3; 10X Genomics) were used to create library samples. Single-cell RNA sequencing was performed using the 10X Genomics Chromium System, which included cell lysis, mRNA reverse transcription, barcoding, UMI incorporation, cDNA amplification, and Illumina HiSeq 4000 sequencing. After excluding cells based on mitochondrial gene count and gene expression variability, cell clustering and the identification of differentially expressed genes were carried out using Harmony for batch correction. Using marker genes identified by differential expression analysis, UMAP displayed clusters after PCA.

### Statistical analysis

GraphPad Prism 9.0 was used for statistical analysis. The levels of GPX4, system Xc^-^, iron, and interferon in primary adipocyte cells and skin tissues were measured using the Student's t-test. The effects of GPX4 on AMT, iron overload, and interferon signaling were evaluated using multiple comparison tests and one- or two-way analysis of variance (ANOVA). When comparing several treatment groups, various treatment pairings, or multiple treatments against a single control, the Benjamini-Hochberg method, Dunnett's test, and Tukey's test were appropriately applied. Two-sided Student's t-tests were used to assess the therapeutic efficacy of the GPX4 inhibitor RSL3, and P-values less than 0.05 were deemed significant.

## Results

### scRNA-seq analysis revealed obvious ferroptosis-resistance in adipocytes of keloid skin tissues

**Figure 1:**
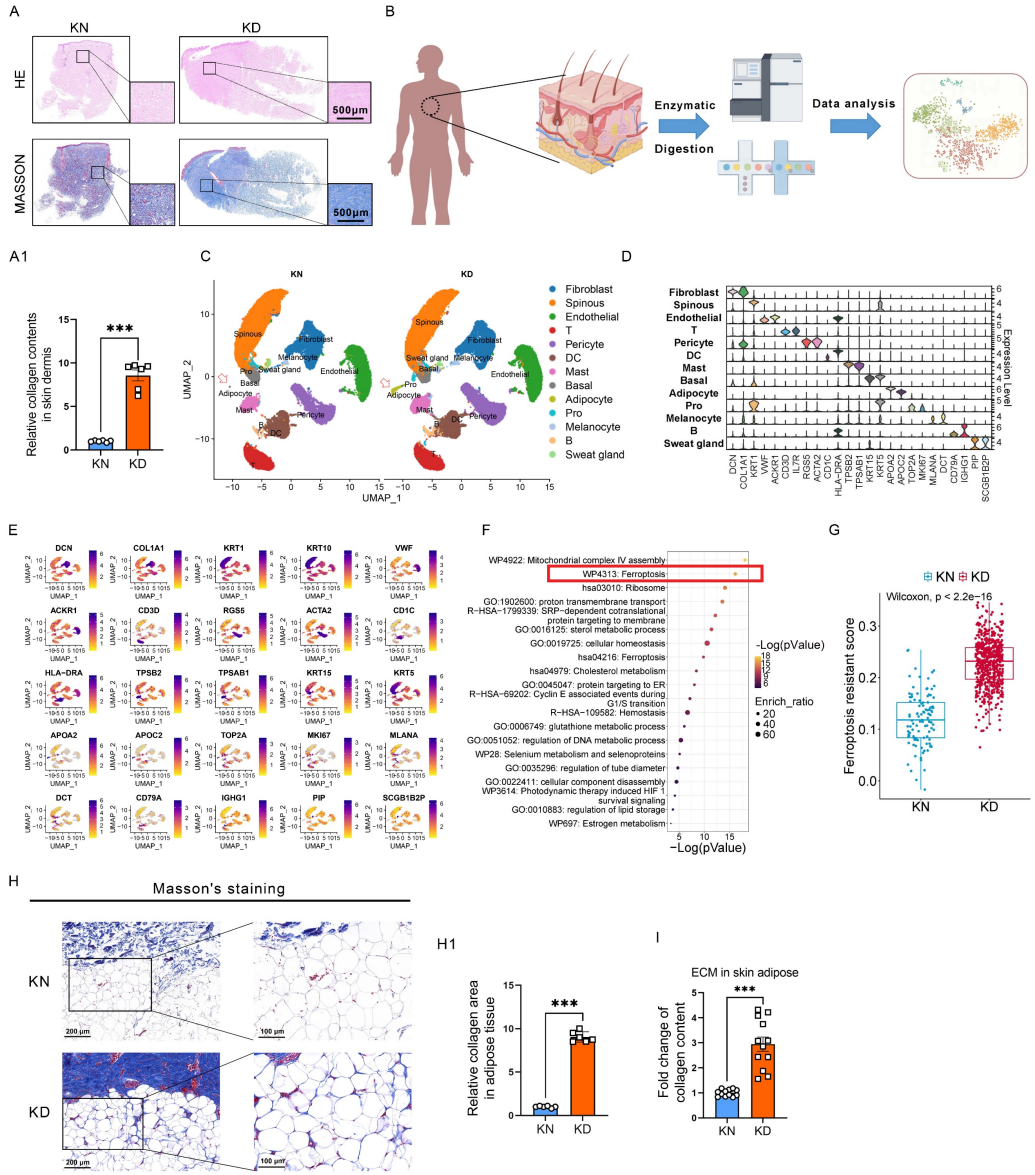
** Significant ferroptosis-resistant characteristics in KD adipocytes. (A-A1)** HE staining, Masson's staining, and collagen content per unit area in KN and KD tissues. Scale bar: 500 μm. **(B)** Workflow for the synthesis, processing, and bioinformatic analysis of KN and KD skin tissues. The 10x Genomics technology is utilized for scRNA-seq analysis. **(C)** UMAP depiction of skin tissue scRNA-seq cell clustering. **(D)** Violin plots of all cell group marker gene expression patterns. **(E)** UMAP visualizations of principal marker genes for thirteen cell types. Purple signifies elevated expression, whilst yellow denotes diminished expression. **(F)** Metascape-based investigation of KD and KN adipocytes' elevated DEGs using scRNA-seq. **(G)** Scores of ferroptosis resistance in adipocytes of KD and KD skin. **(H-H1)** Masson's labeling and measurement of extracellular matrix components in subcutaneous adipose tissue. N = 6 per group. **(I)** Sircol assay of ECM contents in subcutaneous adipose. N = 12 per group. Scale bar, 100 μm and 200 μm. Data in H, I, and J are calculated using the Student's t-test. Data are represented as mean ± SEM. *P < 0.05; **P < 0.01; ***P < 0.001.

Keloids are characterized by alterations in skin morphology, remodeling of dermal structures, and collagen deposition. Hematoxylin & eosin (H&E) and Masson's trichrome staining assays revealed elevated collagen levels in the skin tissues of keloid patients compared to normal skin tissues (**Figure 1A-A1**). We used single-cell RNA sequencing (scRNA-seq) on six pairs of skin biopsies from keloids (KD) and adjacent normal controls (KN) to understand keloids' pathogenesis (**Figure 1B**). Normalization was followed by uniform manifold approximation and projection (UMAP). Twenty-one clusters were identified through established lineage-specific marker genes exclusive to Fibroblasts, Spinous cells, Endothelial cells, T lymphocytes, Peicytes, DC cells, Mast cells, Basal cells, Adipocytes, Melanocytes, B lymphocytes, and Sweat gland cells (**Figure 1C-D**). The UMAP of every cell type with specific markers is shown in (**Figure 1E**). In addition, the relative abundance and distribution of major cell populations are shown in **Supplementary Figure 1**. Next, pathway enrichment analysis was conducted using Metascape, incorporating resources such as the Gene Ontology (GO) and Kyoto Encyclopedia of Genes and Genomes (KEGG), to discover pathways considerably enriched in keloid adipocytes in comparison to normal controls. We discovered that ferroptosis signaling was significantly enriched in the upregulated differential expressed genes (DEGs) of keloid adipocytes (**Figure 1F**). Moreover, the ferroptosis signatures intend to be ferroptosis-resistance, and the score of ferroptosis-resistance was higher in keloid adipocytes than in control adipocytes (**Figure 1G**). The gene panel used to evaluate the ferroptosis resistance levels is listed in **Supplementary Table S1**. These results indicated significant abnormalities in the adipocytes of keloid. Moreover, Masson's staining and Sircol assay of extracellular matrix (ECM) in subcutaneous adipose revealed ECM contents also existed in adipose tissue and were further elevated in keloid patients (**Figure 1H-I**).

These results indicated that ferroptosis resistance of adipocytes is involved in keloid progression and may account for the excessive collagen deposition in keloid.

### Ferroptosis-resistant adipocytes exhibit iron overload and ROS exhaustion

**Figure 2:**
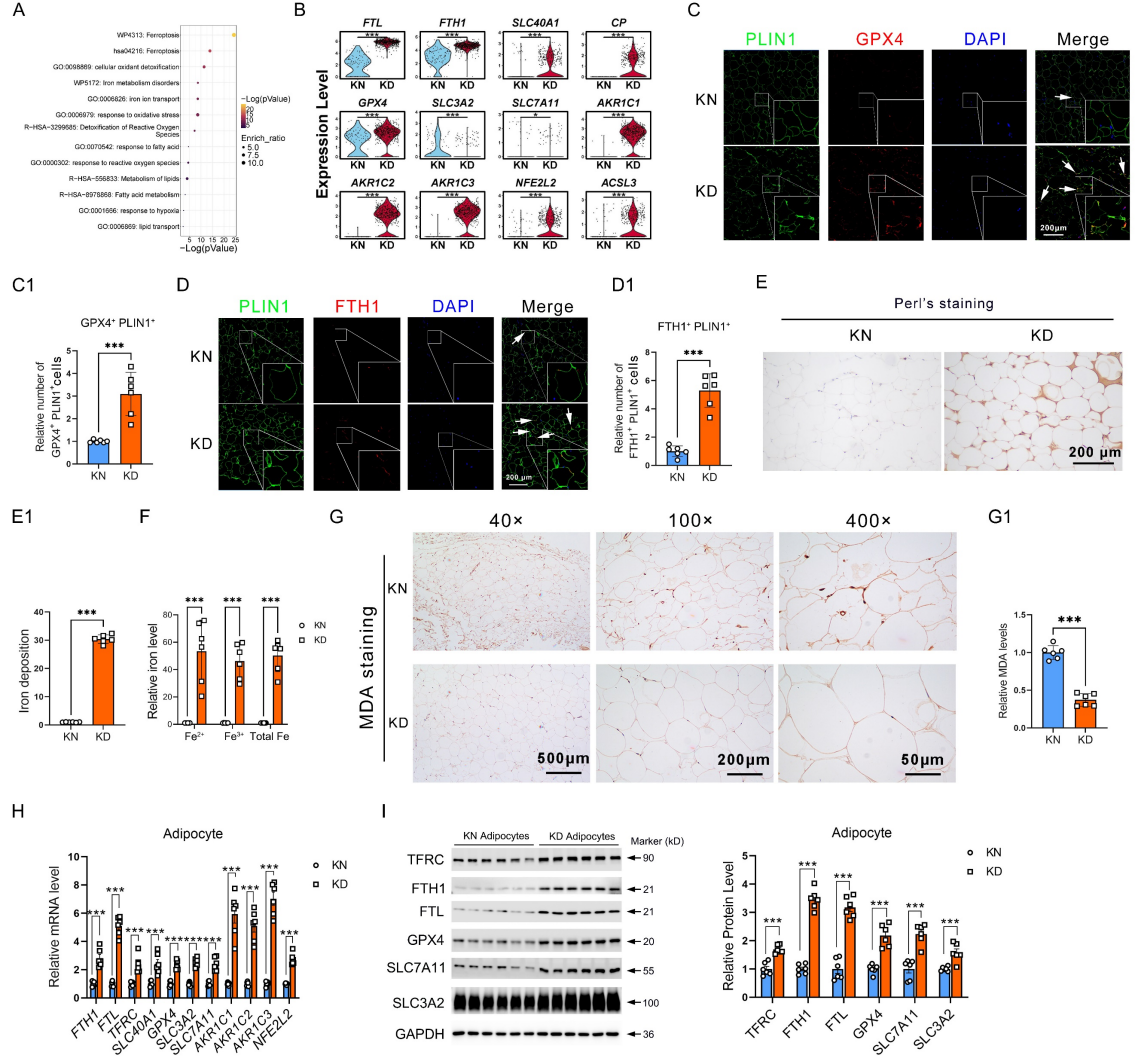
** Excessive iron deposition and ROS exhaustion in KD adipocytes. (A)** Metascape-based analysis of the ferroptosis resistance-related genes, which are enriched in KD adipocytes. **(B)** Violin graphs show iron, ROS, and lipid metabolism gene expression in KN and KD skin-derived adipocyte cells. **(C-C1)** Immunofluorescence test and cell enumeration of PLIN1^+^GPX4^+^ adipocytes in keloid skin specimens. **(D-D1)** Immunofluorescence test and cell quantification of PLIN1^+^FTH1^+^ adipocytes in keloid skin specimens. **(E-E1)** Perl's staining and estimation of iron concentrations in dermal tissues. **(F)** Detection of intracellular iron levels in primary sorted adipocytes from KD and KN tissues. **(G)** Relative MDA levels by IHC assay.** (H-I)** qPCR and western blot examination of KN and KD primary fibroblast iron and ROS metabolism-related protein levels. The scale bar is 200 μm in C**-**E and 500**-**50 μm in G. N=6 per group. Data in B, C1, D1, E1, F, and G are derived from the Student's t-test. The data in H and I are analyzed with the Student's t-test and adjusted using the Benjamin-Hochberg procedure. Data are represented as mean ± SEM. *P < 0.05; **P < 0.01; ***P < 0.001.

Reclustering of the ferroptosis-resistance genes revealed that these resistant genes predominantly participate in iron metabolism, lipid metabolism, and ROS metabolism, suggesting that ferroptosis-resistance was triggered by these abnormal pathways in KD (**Figure 2A**). Further, we showed the representative genes in each key event by scRNA-seq. We found that solute carrier family 40 member 1 (*SLC40A1*, also referred to as *FPN1*), ferritin heavy chain 1 (*FTH1*), ferritin light chain (*FTL*), and ceruloplasmin (*CP*) for iron metabolism, glutathione peroxidase 4 (*GPX4*), aldo-keto reductase family 1 member C (*AKR1Cs*), system Xc^-^ members solute carrier family 3 member 2 (*SLC3A2*), solute carrier family 7 member 11 (*SLC7A11*), and NFE2 Like BZIP Transcription Factor 2 (*NFE2L2*) for ROS metabolism, along with Acyl-CoA Synthetase Long Chain Family Member 3 (*ACSL3*) for lipid metabolism were all significantly upregulated in KD adipocytes (**Figure 2B**). Immunofluorescence staining showed increased PLIN1^+^GPX4^+^ and PLIN1^+^FTH1^+^ cells in KD adipocytes (**Figure 2C-D**). Subsequently, we conducted Perl's staining and observed elevated iron concentrations in KD adipocytes relative to control adipocytes, indicating iron overload in KD adipocytes (**Figure 2E-E1**). Detection of iron content revealed elevated intracellular iron levels in primary adipocytes from KD tissues in comparison to KN tissues (**Figure 2F**). IHC also revealed that the MDA, a ferroptosis marker, decreased in KD adipocytes (**Figure 2G**). We evaluated the expression of iron and ROS metabolism genes in primary sorted adipocytes to confirm that KD-derived adipocytes have impaired iron metabolism and resist ferroptosis. Iron metabolism genes TFRC, FTH1, SLC40A1, FTL, and oxidative stress-related genes GPX4, SLC3A2, SLC7A11, AKR1Cs, and NFE2L2 were considerably enhanced at both mRNA and protein levels (**Figure 2H-I**). In addition, iron overload, excessive ROS clearance, and a corresponding dysregulation of genes were also found in the KD skins (**Supplementary Figure 2**). These results highly support the idea that the adipocytes of keloid are ferroptosis-resistant.

We have shown that ferroptosis-resistant adipocytes in keloids are subject to iron overload and ROS exhaustion.

### Activation of GPX4 endows ferroptosis-resistance properties and induces AMT in adipocytes

**Figure 3:**
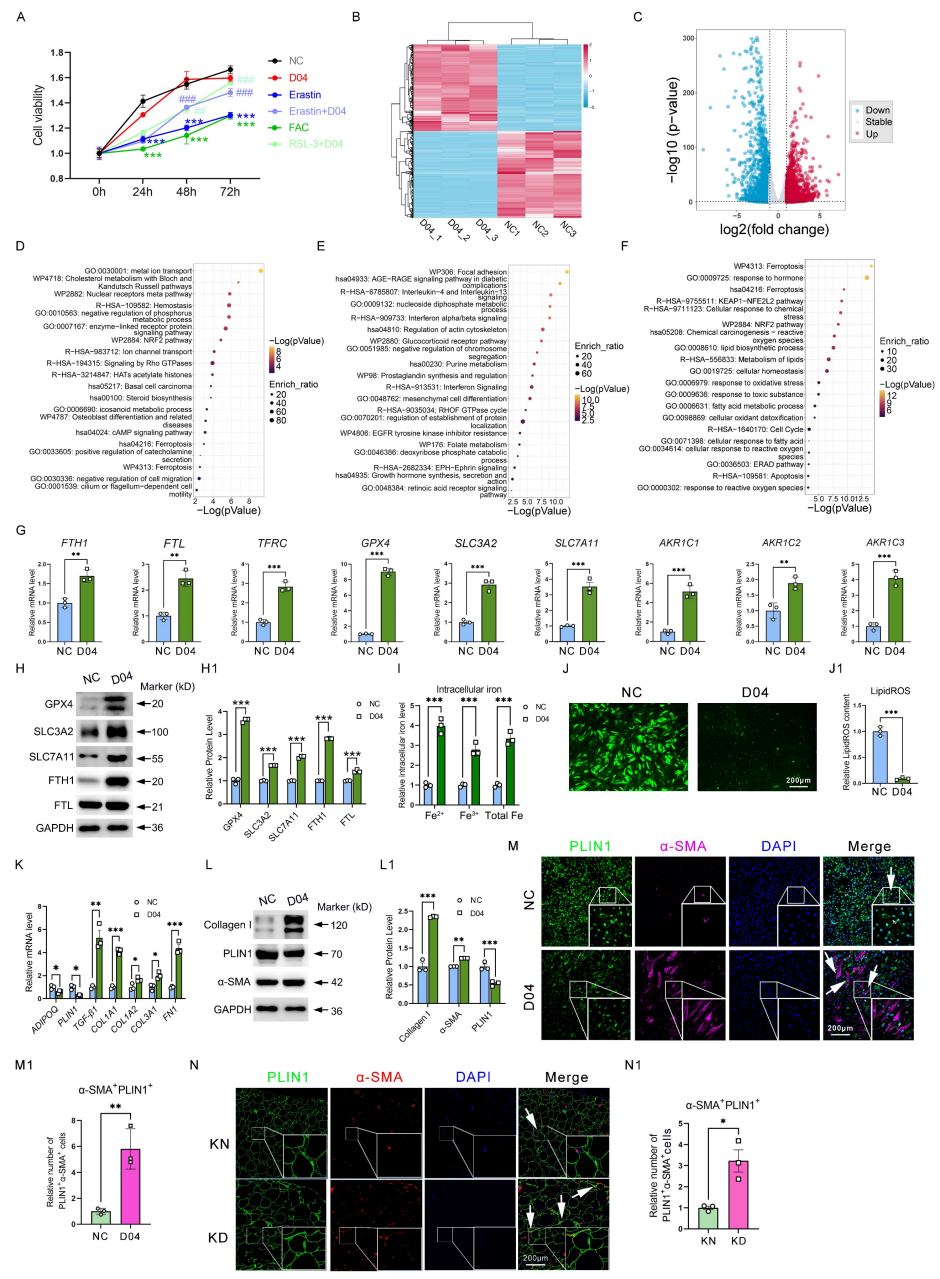
** GPX4 induces iron overload, ROS exhaustion, and AMT in adipocyte cells. (A)** Cell viability of adipocytes with D04, Erastin, Erastin+D04, FAC, and FAC+D04 treatments. **(B-C)** The DEG heatmap and volcano plot between control and D04-treated fibroblasts. **(D-E)** The upregulated and downregulated DEGs upon D04 treatment were annotated based on functional enrichment results from Metascape, respectively. **(F)** D04-treated and KD adipocytes shared common enriched functional categories identified by Metascape. **(G)** mRNA levels of iron metabolism and glutathione metabolism genes. **(H)** Protein levels of GPX4, SLC3A2, SLC7A11, FTH1 and FTL in D04-treated adipocytes. **(I)** Intracellular levels in adipocytes after D04 treatment. **(J)** LipidROS levels in adipocytes after D04 treatment. **(K-L)** Levels of mRNA and protein for adipocyte and fibroblast cell markers. **(M-M1)** Immunofluorescence test and cell enumeration of PLIN1^+^α-SMA^+^ adipocytes. **(N-N1)** Immunofluorescence test and cell quantification of PLIN1^+^α-SMA^+^ adipocytes in keloid skin tissues. N = 3 per group. The scale bar is 200 μm in M and N. The data is evaluated using two-way ANOVA and the Tukey test. The Student's t-test yields G, I, J, M1, and N1 data. Student's t-test and Benjamini-Hochberg adjustment are used for H, K, and L data. Data are presented as mean ± SEM. Three replicates are done for all experiments. *P < 0.05; **P < 0.01; ***P < 0.001 compared. controls. Compared to different treatments, #P < 0.05, #P < 0.01; #P < 0.001.

Considering that GPX4 has elevated levels in ferroptosis-resistant adipocytes and is one of the most critical proteins controlling ferroptosis. Therefore, we added a GPX4 agonist PKUMDL-LC-101-D04 (D04) into adipocytes *in vitro* to mimic the ferroptosis-resistance phenomenon observed *in vivo*. It was found that D04 significantly mitigated the decrease in cell viability induced by Erastin and FAC, two ferroptosis inducers, suggesting that GPX4 could inhibit ferroptosis (**Figure 3A**). Furthermore, we detected changes in the whole transcriptomics of adipocytes after D04 treatment by RNA-seq. There was a noticeable difference between the PBS control group and the D04 groups in the gene expression profile heatmap (**Figure 3B**).

Finally, 4,127 DEGs (Fold change (FC) ≥ |1.0|, P < 0.05) were identified, including 1,998 upregulated genes and 2,129 downregulated genes (**Figure 3C**). Metascape-based enrichment analysis revealed that the upregulated DEGs by D04 were majorly involved in metal ion transport, NRF2 pathway, ferroptosis, and so on (**Figure 3D**). In addition, the downregulated DEGs by D04 were majorly involved in focal adhesion, interleukin-4/13, and interferon pathways (**Figure 3E**). Venn analysis and enrichment analysis of upregulated genes in D04-treated adipocytes with upregulated genes in KD adipocytes by scRNA-seq analysis revealed that the ferroptosis, KEAP1-NFE2L2, and NRF2 pathways were still enriched, and these genes were associated with iron overload and ROS exhaustion (**Figure 3F**). qPCR results verified that genes such as *FTH1, FTL, TFRC, GPX4, SLC3A2, SLC7A11,* and *ACKRs* were significantly upregulated by GPX4 in adipocytes (**Figure 3G**). Western blot further confirmed that these genes were also significantly upregulated at the protein level (**Figure 3H**). In addition, intracellular iron levels were increased in the D04 group, while LipidROS levels were significantly decreased (**Figure 3I-J**). These results suggest that activation of GPX4 effectively recapitulated the ferroptosis resistance, iron overload, and ROS exhaustion observed in adipocytes of KD patients. More importantly, we found that D04 treatment significantly induced collagen expression in adipocytes, which was realized as a significant increase in fibrotic genes and proteins such as TGF-β1, FN1, collagens, and α-SMA, consistent with the increased collagen in adipose tissue sites seen in Figure 1. In addition, we found that GPX4 resulted in the loss of adipocyte-specific markers, including ADIPOQ and PLIN1 (**Figure 3K-L**). Immunofluorescence analysis further validated increased α-SMA-positive adipocytes with D04 treatment (**Figure 3M**). These results suggested that GPX4 elicited an adipocyte-mesenchymal transition (AMT) process. Staining of KD tissue also showed that AMT accumulated in KD adipocytes (**Figure 3N**).

### GPX4 activates canonical and non-canonical TGF-β signaling to induce AMT through iron overload

**Figure 4:**
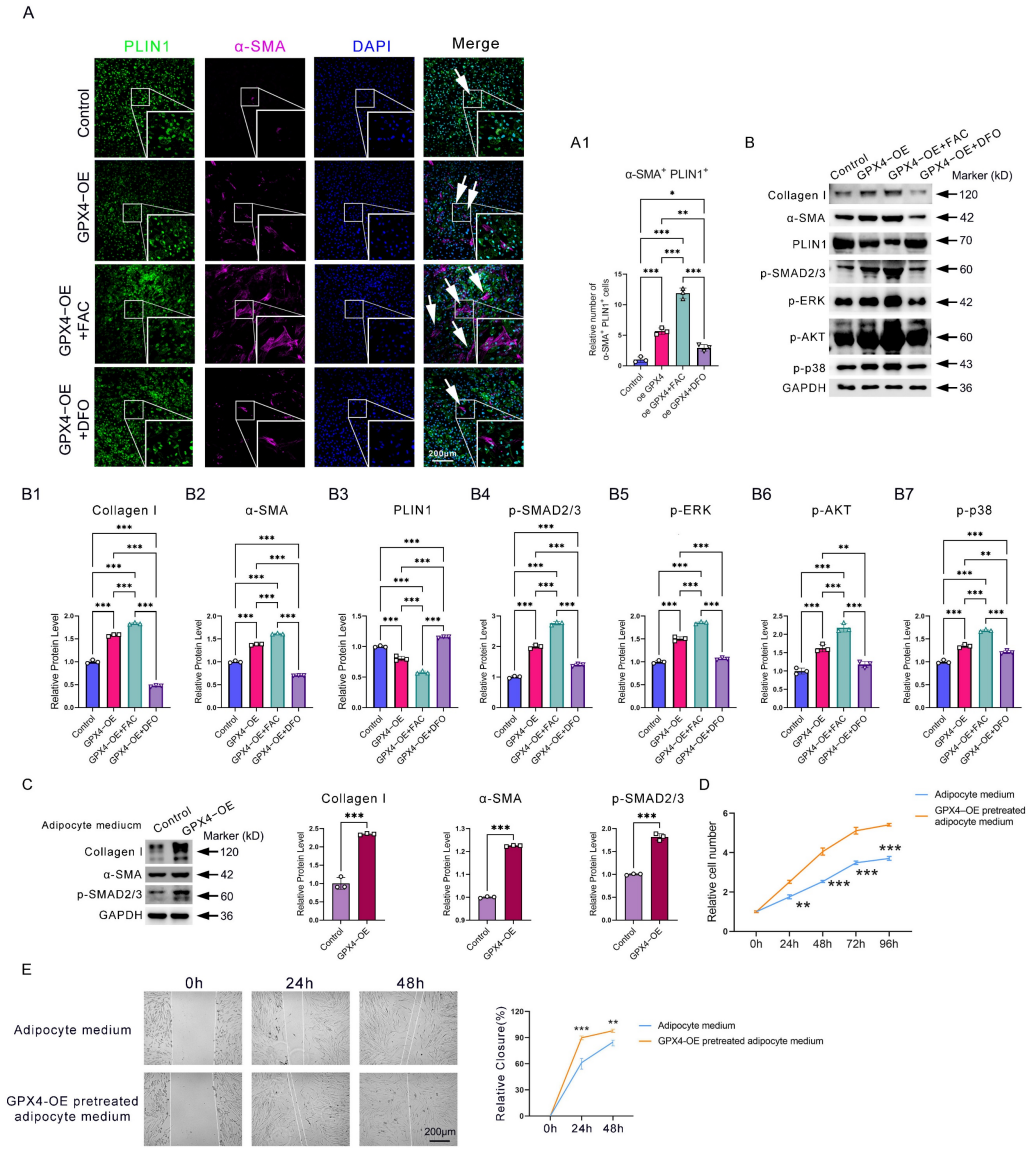
** GPX4 promotes iron deposition and activates the TGF-β pathway to induce AMT eventually. (A-A1)** Immunofluorescence assay and cell count of PLIN1^+^α-SMA^+^ adipocytes in GPX4**-**OE, GPX4**-**OE+FAC, GPX4-OE+DFO groups. Scale bar: 200 μm. **(B)** Western blot examination and quantification of Collagen I, α-SMA, p-AKT, p-SMAD2/3, p-ERK1/2, and PLIN1 in adipocytes. **(C)** Western blot analysis of fibrosis-associated proteins in KD fibroblast cells treated with either control adipocyte cell media or GPX4-OE-pretreated adipocyte cell medium. **(D-E)** Cell proliferation and migration of KD fibroblast cells pretreated with adipocyte cell medium, with or without GPX4-OE treatment. The data in A and B1-B7 are analyzed using one-way ANOVA with the Tukey test. The Student's t-test assesses data in C, D, and E. Data are expressed as mean ± SEM. All tests are conducted in triplicate. *P < 0.05; **P < 0.01; ***P < 0.001.

Next, we examined the mechanism responsible for the accumulation of AMT in GPX4-mediated ferroptosis-resistant adipocytes. Since iron overload is an essential feature in GPX4-induced ferroptosis-resistant adipocytes, we then examined the effect of iron on AMT. Immunofluorescence analysis showed that iron supplementation with ferric citrate (FAC) in adipocyte cells significantly enhanced GPX4 overexpression (GPX4-OE)-induced AMT. In contrast, deferoxamine (DFO) iron chelation significantly inhibited GPX4-induced AMT (**Figure 4A-A1**). Western blotting analysis further confirmed that GPX4 increased FAC-induced α-SMA and collagen I expression in adipocytes while decreasing PLIN1 expression (**Figure 4B, B1-B3**). These results indicate that AMT is dependent on iron overload.

To explain why iron overload induces AMT, we examined the activities of TGF-β pathways. TGF-β is widely involved in AMT, endothelial-mesenchymal transition (EndoMT), epithelial-mesenchymal transition (EMT), and collagen deposition. Western blot showed increased levels of p-SMAD2/3, p-AKT, p-ERK1/2, and p-p38 in the GPX4-OE group relative to the control group (**Figure 4B, B4-B7**). To evaluate the impact of AMT on adipocytes in the context of fibrosis, we prepared a conditional medium from adipocyte cells pretreated with GPX4-OE or PBS. Results showed that conditional medium from GPX4 overexpression-pretreated adipocyte cells significantly promoted the production of the fibrotic proteins α-SMA and collagen I, along with the activation of the TGF-β/SMAD2/3 fibrotic pathways (**Figure 4C**). In addition, conditioned media from GPX4-pretreated adipocytes similarly enhanced the rate of fibroblast migration and proliferation (**Figure 4D-E**).

The data demonstrate that GPX4 activates AMT via both canonical and non-canonical TGF-β signaling pathways triggered by iron overload.

### Downregulation of interferon signaling in KD adipocytes caused by GPX4-induced iron overload and ROS exhaustion strengthen adipocyte AMT and fibroblast fibrosis

**Figure 5:**
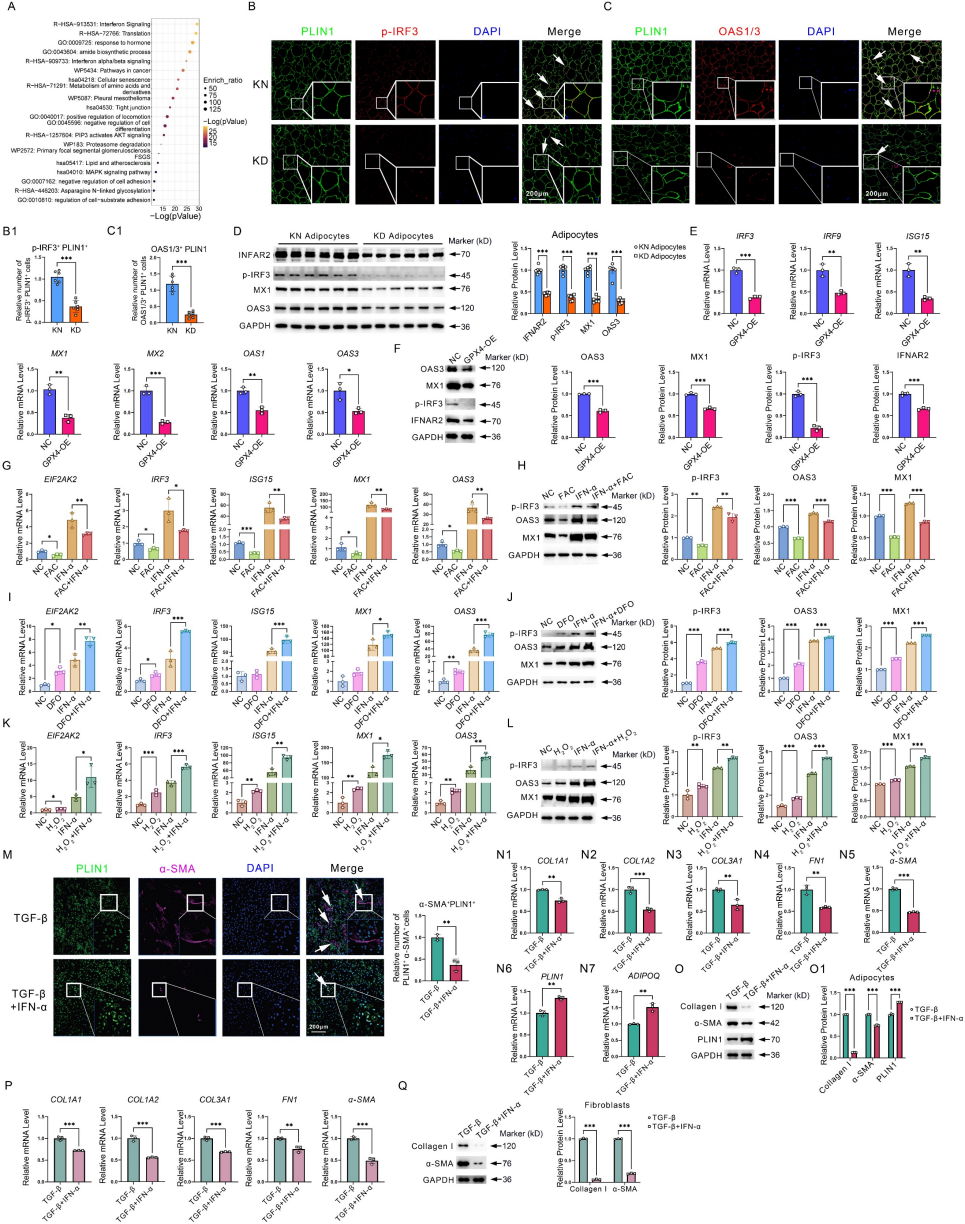
** GPX4 inhibits interferon signaling in adipocyte cells. (A)** Metascape-based functional enrichment of downregulated DEGs in KD adipocytes identified by scRNA-seq. **(B-C)** Immunofluorescence and cell count of PLIN1^+^p-IRF3^+^ and PLIN1^+^OAS1/3^+^ adipocytes. N = 3 per group. Scale bar: 200 μm. **(D)** IFNAR2, p-IRF3, MX1, and OAS3 protein levels in KD and KN adipocytes. N = 6 per group. **(E)** mRNA levels of interferon-related genes in GPX4-OE-treated adipocytes. **(F)** Protein levels of IFNAR2, p-IRF3, MX1, and OAS3 in GPX4-OE-treated adipocytes. **(G-H)** mRNA and protein levels of interferon-related genes adipocytes after interferon and/or FAC treatments. **(I-J)** mRNA and protein levels of interferon-related genes adipocytes after interferon and/or DFO treatments. **(K-L)** mRNA and protein levels of interferon-related genes adipocytes after interferon and/or H_2_O_2_ treatments. **(M)** Immunofluorescence assay and cell count of PLIN1^+^α-SMA^+^ adipocytes. N = 3 per group. Scale bar: 200 μm. **(N1-N7)** mRNA levels of fibrotic and adipocyte cell genes in adipocytes. **(O-O1)** Western blot analysis and quantification of Collagen I, α-SMA, and PLIN1 in adipocytes. **(P-Q)** qPCR and western blot analysis of fibrotic genes and proteins in fibroblast cells. The Student's t-test assesses data in B, C, E, F, M, N, and P. The Student's t-test evaluates data in D, G, H, I, J, K, L, O1, and Q, which is subsequently adjusted using the Benjamini-Hochberg process. All experiments are repeated in triplicate. Data are represented as mean ± SEM. *P < 0.05; **P < 0.01; ***P < 0.001 *vs.* controls.

We further enriched and analyzed the downregulated DEGs in KD adipocytes and found that interferon-related genes were drastically reduced (**Figure 5A**). Double immunofluorescence staining showed decreased PLIN1^+^p-IRF3^+^(**Figure 5B-B1**) and PLIN1^+^OAS1/3^+^ (**Figure 5C-C1**) cells in KD adipocytes. Western blot revealed lower phosphorylated IRF-3, IFNAR2, MX1, and OAS3 levels in KD adipocytes than in KN adipocytes (**Figure 5D**). Similar results were also observed in KD skin tissues compared to normal control skins (**Supplementary Figure 3**). Next, we detected the activity of the interferon pathway in GPX4 overexpression-induced ferroptosis-resistant adipo-cytes. As shown in Figure 3E, interferon pathways were significantly enriched in GPX4 downregulated DEGs. qPCR analysis confirmed that *IRF9*, *IRF3*, *ISG15*, *MX1*, *MX2*, *OAS1*, and *OAS3* were all decreased in GPX4-OE adipocytes compared to control adipocytes. In addition, the western blot showed that GPX4 significantly inhibited OAS3, MX1, p-IRF3, and IFNAR2 at protein levels (**Figure 5E-F**). These results implicated ferroptosis-resistant adipocytes associated with impaired interferon signaling. Since ferroptosis-resistant adipocytes have significant iron overload and ROS depletion, we hypothesized that the cause of interferon non-response due to ferroptosis resistance is related to these two events. We first treated adipocytes with FAC and showed that FAC inhibited interferon-related gene and protein expression with or without IFN-α2 stimulation (**Figure 5G-H**). The iron chelator DFO consistently upregulated interferon-related gene and protein expression (**Figure 5I-J**). Besides, hydrogen peroxide (H_2_O_2_), which increases cellular free reactive oxygen species (ROS), significantly increased interferon-related expression with or without IFN-α2 stimulation (**Figure 5K-L**). These results suggest that interferon inhibition due to ferroptosis resistance is dependent on iron overload and ROS exhaustion.

Given its significantly low expression in KD, what role does interferon play in KD? Thus, we examined the role of interferon in adipocyte as well as fibroblast fibrosis. Immunofluorescence staining revealed that interferon significantly inhibited TGF-β-induced AMT in adipocytes, as evidenced by a substantial decrease in the quantity of PLIN1^+^α-SMA^+^ cells (**Figure 5M**). In addition, qPCR and western blot experiments revealed that interferon significantly inhibited fibrosis-related genes and protein expression, such as collagen, yet promoted adipocyte markers ADIPOQ and PLIN1 expression (**Figure 5N-O**). In addition, we further found that interferon significantly inhibited fibroblast fibrotic response (**Figure 5P-Q**). These results suggest that interferon signaling pathway decay would, on the one hand, enhance AMT and induce collagen synthesis in adipocytes. On the other hand, interferon signaling pathway impairment would lead to uncontrolled collagen synthesis in fibroblasts. The synergistic effects of interferon in adipocytes and fibroblasts ultimately lead to excessive collagen synthesis, thereby exacerbating KD.

### GPX4 overexpression in adipocytes activated fibroblast by transferring iron and Fibroblast-derived cystine sustains high activity of ferroptosis resistance of adipocytes

**Figure 6:**
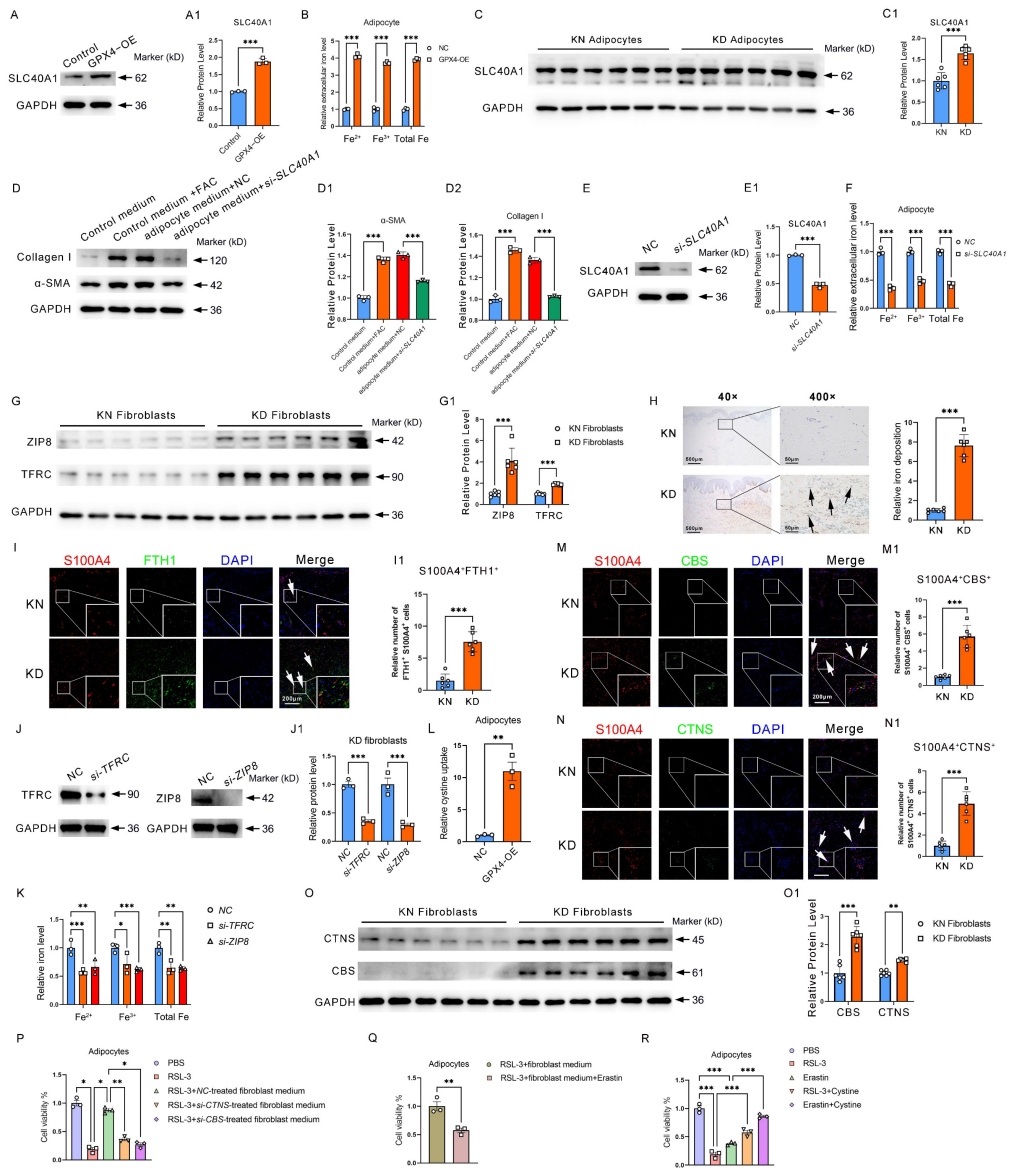
** GPX4-mediated adipocyte-fibroblast cell communication enhances ferroptosis resistance in adipocyte and fibroblast collagen synthesis via iron-cystine exchange. (A)** Protein levels of SLC40A1 in GPX4-OE adipocytes. **(B)** Relative extracellular iron levels in GPX4-OE adipocytes. **(C)** Protein levels of SLC40A1 in KD and KN adipocytes. **(D)** Levels of Collagen I and α-SMA in fibroblast cells after control medium with or without FAC treatments, or treated with a medium derived from adipocytes which pretreated with or without *si-SLC40A1*. **(E)** Protein levels of SLC40A1 in *si-SLC40A1* adipocytes. **(F)** Relative extracellular iron levels in *si-SLC40A1* adipocytes. **(G)** Protein levels of ZIP8 and TFRC in KD and KN fibroblasts. **(H)** Skin tissue iron assessment by Perl's staining. **(I-I1)** Assay for immunofluorescence and S100A4^+^FTH1^+^ adipocyte cell count. N = 3 per group. Scale bar: 200 μm. (J) Protein levels of TFRC and ZIP8 in fibroblasts. **(K)** Relative intracellular iron levels in GPX4-OE fibroblasts. **(L)** Relative ability of cystine uptake in GPX4-OE adipocytes. **(M-N)** CTNS and CBS protein levels in KD and KN fibroblasts by immunofluorescence staining. **(O)** CTNS and CBS protein levels in KD and KN fibroblasts by western blot analysis. **(P-R)** Cell viability of adipocytes. The Students' t-test evaluates data in A-C and E-O. Data in D is evaluated using the Student's t-test and corrected using the Benjamin-Hochberg procedure. Data in P-R are evaluated by one-way ANOVA (Tukey test). All experiments are repeated in triplicate. Data are represented as mean ± SEM. *P < 0.05; **P < 0.01; ***P < 0.001 *vs.* controls.

We have found that GPX4 overexpression induced adipocyte iron deposition. Interestingly, we found that GPX4 significantly induced adipocyte SLC40A1 expression and increased extracellular iron levels (**Figure 6A-B**). As shown in Figure 3I, intracellular iron levels were also upregulated by GPX4, suggesting that iron efflux was enhanced in GPX4-treated adipocytes. Elevation of SLC40A1 expression was thus detected in adipocytes from KD patients (FC = 1.645, P < 0.001) (**Figure 6C**). Next, we evaluated the impact of adipocytes on fibroblast activation and found that medium from GPX4-overexpressed adipocytes significantly promoted fibroblast collagen synthesis (**Figure 6D-D2**). To further determine the role of iron in this promotion, we used* si-SLC40A1* to inhibit iron efflux from adipocytes. The findings indicated that *si-SLC40A1* markedly reduced the extracellular iron levels in adipocytes (**Figure 6E-E1**). Moreover, the adipocyte cell culture medium after *si-SLC40A1* lost its promotional effect on fibroblast collagen synthesis (**Figure 6D-D2**). In contrast, the addition of FAC to fibroblasts significantly promoted fibroblast collagen synthesis (**Figure 6D-D2**). It is well known that iron uptake is mediated through receptors. Thus, we examined the expression levels of iron receptors TFRC and solute carrier family 39 member 8 (SLC39A8, also known as ZIP8) in KD fibroblasts. It was found that the expression of iron receptors expression was increased in KD fibroblasts compared to KN (TFRC: FC = 1.893, P < 0.001; ZIP8: FC = 4.099, P < 0.001) (**Figure 6G-G1**). Perl's staining also indicated significant iron overload at the KD dermis (**Figure 6H**). Immunofluorescence experiments revealed that FTH1^+^S100A4^+^ cells were significantly increased in KD tissues, indicating iron overload in KD fibroblasts (**Figure 6I-I1**). Moreover, the knockdown of TFRC and ZIP8 significantly decreased intracellular iron levels in KD fibroblasts (**Figure 6J-K**). These results demonstrated that GPX4-overexpressing adipocytes could deliver iron extracellularly via elevated expression of the iron efflux protein SLC40A1 and that fibroblasts increased the iron receptors to take up adipocyte-derived iron to promote their malignant hyperplasia and collagen deposition.

Cystine is an important substrate for synthesizing glutathione and one of the key metabolites for maintaining GPX4 activity. We have found that GPX4 expression is increased in KD adipocytes. More interestingly, we found that the cystine receptor system Xc^-^ was also significantly increased (Figure 2I), suggesting that KD adipocytes require extracellular cystine uptake to maintain high GPX4 activity and subsequently obtain ferroptosis-resistance properties. In addition, we further found significantly enhanced cystine uptake in GPX4**-**OE adipocytes (**Figure 6L**). A key question is, where does cystine come from? We found that protein levels of CBS and CTNS, two proteins related to cystine synthesis and efflux, respectively, were significantly elevated in KD primary fibroblasts compared to normal human skin fibroblasts by immuno-fluorescence staining (**Figure 6M-N**). Western blot further showed that CBS and CTNS were considerably elevated in KD fibroblasts (**Figure 6O-O1**). These results suggest that KD fibroblasts have a significantly improved ability to deliver cystine externally.

To further determine the role of fibroblasts in resistance to ferroptosis in adipocytes, we carried out cell cross-talk experiments. The results revealed that fibroblast medium significantly inhibited RSL3-induced ferroptosis in adipocytes (**Figure 6P**). However, when CTNS was knocked down in fibroblasts, the fibroblast culture medium could no longer inhibit RSL3-induced ferroptosis in adipocytes (**Figure 6P**). The promotion of ferroptosis resistance in adipocytes by fibroblast culture medium was similarly blocked by treating adipocytes with the system Xc^-^ inhibitor Erastin (**Figure 6Q**). In addition, the direct addition of cystine to adipocytes alleviated both RSL3- and Erastin-induced ferroptosis (**Figure 6R**). These results suggest that fibroblasts maintain adipocyte ferroptosis resistance by delivering cystine to adipocytes.

### Inhibition of GPX4 or blockage of GPX-induced iron overload improved keloid *in vivo*

**Figure 7:**
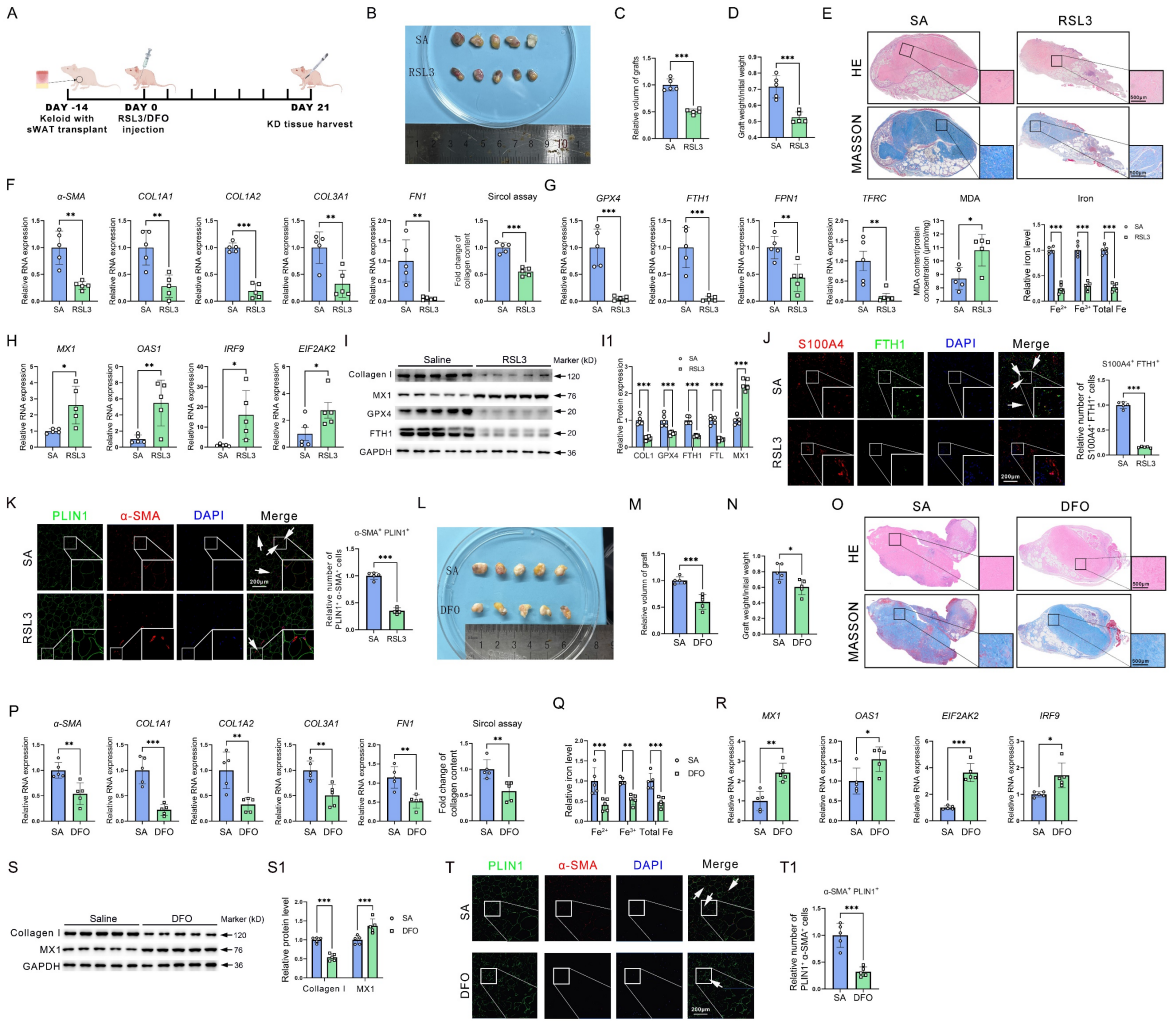
** Inhibition of GPX4 by RSL3 alleviates keloid in mice. (A)** RSL3 is injected subcutaneously into the mouse model of keloid-bearing mice. **(B-D)** Measurement of graft volume and weight. **(E)** HE and Masson staining the harvested grafts. **(F)** Fibrosis-related gene detection and Sircol assay of ECM in saline- and RSL3-injected grafts. **(G)** Measurements of MDA and iron levels and mRNA levels of *GPX4*, *FTH1*, *SLC40A1*, and *TFRC* in KD grafts. **(H)** mRNA levels of interferon-related genes in KD grafts. **(I-I1)** Protein levels of Collagen I, MX1, GPX4, and FTH1 in KD grafts. **(J-K)** Immunofluorescence test and quantification of S100A4^+^FTH1^+^ fibroblast cells and PLIN1^+^α-SMA^+^ adipocyte cells. **(L-N)** Measurement of graft volume and weight. **(O)** HE and Masson stain the harvested grafts. **(P)** Fibrosis-related gene detection and Sircol assay of ECM in saline- and DFO-injected grafts. **(Q)** Measurements of iron levels in KD grafts. **(R)** mRNA levels of interferon-related genes in KD grafts. **(S-S1)** Collagen I and MX1 protein levels in KD grafts. **(T-T1)** Immunofluorescence assay and quantification of PLIN1^+^α-SMA^+^ adipocyte cells. All scale bars are 200 μm. N = 5 per group. The Student's t-test assesses data in A-H and J-K. The data in I is assessed by the Student's t-test and adjusted using the Benjamini-Hochberg procedure. Data are represented as mean ± SEM. *P < 0.05; **P < 0.01; ***P < 0.001 *vs.* controls.

Given the promotion of ferroptosis resistance on iron overload, AMT, and collagen deposition, we examined the potential of reinstating ferroptosis to mitigate keloid formation *in vivo*. Grafts were taken on day 24 after the keloid-bearing animal had repeated injections of PBS or RSL3 every three days (**Figure 7A**). In comparison to the NC group, subcutaneous keloid grafts showed decreased weight and volume (**Figure 7B-D**). Histological analysis indicated that RSL3 therapy decreased the density of cutaneous tissue (**Figure 7E**). RSL3 injection into keloid tissue dramatically decreased the mRNA levels of fibrotic genes, such as *α-SMA, COL1A1, COL1A2, COL3A1,* and *FN1,* and downregulated the ECM contents, according to qPCR analysis and the Sircol test (**Figure 7F**). Next, we set out to investigate the mechanisms underlying RSL3-mediated collagen reduction. qPCR showed a reduction in GPX4, an elevation of MDA levels, and downregulation of iron overload-related genes and iron levels in RSL3 groups (**Figure 7G**). Additionally, interferon-related genes* MX1, OAS1, IRF9* and *EIF2AK2* were upregulated by RSL3 as well (**Figure 7H**). Western blot showed a reduction in collagen I, GPX4, and FTH1, while MX3 levels in RSL3 groups increased (**Figure 7I-I1**). Immunofluorescence revealed the presence of RSL3 in these grafts, alongside a reduction in S100A4^+^FTH1^+^ fibroblast cells and PLIN1^+^α-SMA^+^ adipocyte cells in keloid grafts following RSL3 therapy (**Figure 7J-K**), consistent with *in vitro* experiments. Since iron overload was a significant effect induced by GPX4, we further evaluated the role of inhibiting iron overload in alleviating KD. The results revealed that KD was significantly attenuated after reducing iron content in the KD grafts using DFO, as supported by the assessments of weight, volume, and histopathological changes of KD grafts, as well as collagen content assay, interferon signaling pathway, and AMT detection in KD grafts (**Figure 7L-T**).

These results suggested that the inactivation of GPX4 could restore the sensitivity of ferroptosis and interferon signaling, release iron overload, and eventually alleviate keloid progression* in vivo*.

## Discussion

Adipocytes serve as the principal energy storage cells in the body, facilitating the accumulation and release of fat 4. Subcutaneous adipocytes are stored in the adipose tissue beneath the skin, below the dermis, and above the deep fascial layer wrapped by the superficial fascia. About 2/3 of the body's adipose tissue is located in the subcutaneous layer 24. In addition, adipocytes are involved in the secretion of hormones, such as leptin and growth hormone, which affect appetite, metabolism, and energy expen-diture 25. Adipocyte abnormalities are linked to a number of metabolic disorders, such as diabetes, insulin resistance, and obesity 4. In recent years, subcutaneous adipocyte abnormalities have attracted increasing attention as a cause of skin diseases such as seborrhea 26. Subcutaneous adipocyte abnormalities have also been found to be involved in fibrotic skin diseases such as scleroderma 6, fibrolipoma^27^, and sclerosing panniculitis 28. However, the existence of subcutaneous adipocyte abnormalities in KD and their role and mechanism in the development of KD are still unclear.

Through single-cell RNA-seq research, we first found that KD-derived adipocytes have a strong antioxidant capacity and substantially higher ferroptosis resistance scores than those in control KN tissues. In addition, we found that in addition to the dermis, the extracellular matrix contents in the KD adipose layer were considerably elevated compared to those in the KN group, suggesting an underappreciated role of adipocytes in regulating ECM deposition. Next, we clustered the ferroptosis resistance genes enriched in KD to explore further the mechanisms behind ferroptosis resistance in adipocytes. The results revealed that oxidative stress, iron metabolism, and lipid metabolism re-programming were extensively involved, suggesting that KD adipocyte ferroptosis resistance is co-regulated by multiple signaling pathways. This series of adipocyte abnormalities and their roles and mechanisms in fibrogenesis that we have identified in keloids will also contribute to unraveling other skin fibrosis pathogenic mechanisms. For example, during the pathogenesis of SSc, the fat layer is significantly lost 5. An in-depth concern about the role of iron death, iron overload, interferon, and GPX4 expression in SSc adipocytes will also likely be a potential intervention strategy for SSc. Notably, although iron overload is thought to promote ferroptosis, KD adipocytes exhibited ferroptosis resistance properties associated with its high internal expression of GPX4. GPX4 is a powerful ferroptosis suppressor gene, so KD adipocytes can have both ferroptosis resistance and iron overload characteristics. Certainly, it may also be related to the fact that adipocytes have a lower ferroptosis sensitivity. For example, erythrocytes, macrophages, and fibroblasts also have high internal iron content but are not susceptible to ferroptosis 29. Interestingly, iron overload in KD adipocytes is not purely intracellular iron retention but rather a simultaneous enhancement of iron uptake, retention, and efflux. Differences in intracellular and extracellular iron flow rates may have ultimately contributed significantly to the iron overload in KD adipocytes.

Considering that GPX4 is significantly expressed in KD adipocytes and serves as one of the most critical ferroptosis protective factors, we then explored whether we could replicate adipocytes' ferroptosis resistance properties in KD patients by activating GPX4. It was found that the use of GPX4 agonist PKUMDL-LC-101-D04 or overexpression of GPX4 not only successfully induced adipocyte ferroptosis resistance but also led to the occurrence of iron overload in the cells, suggesting that GPX4 is an essential factor leading to iron accumulation and ferroptosis resistance in KD adipocytes. In addition, we found that adipocytes could synthesize collagen, consistent with the finding that collagen deposition also occurred in adipocytes, as revealed by KD Masson staining in Figure 1. More importantly, GPX4 significantly enhanced adipocyte collagen synthesis, and AMT occurred. Mechanistically, this was associated with GPX4-induced iron overload. Chelation of iron using DFO then significantly blocked GPX4-induced AMT and collagen deposition. In previous reports, AMT was also found to occur in subcutaneous adipocytes 7. Here, we further revealed that the causative factor for AMT occurrence was iron overload due to high GPX4 expression. In gastric cancer cells, GPX4 enhances EMT and metastasis by depleting ROS and inhibiting ferroptosis 30, further suggesting an important facilitating role of GPX4 in cellular mesenchymal-like transformation. Currently, GPX4 inhibitory means (BBP-954, DC-2, and DMOCPTL) have been recognized as possible therapeutic approaches for addressing cancer progression and metastasis, and relevant preclinical experiments are underway 31-33. These therapies targeting GPX4 will also positively contribute to the treatment of KD.

In addition to cellular iron overload and ferroptosis resistance, GPX4 leads to impaired interferon signaling in adipocytes. In KD patients, we similarly found significantly decreased expression of several interferon-related genes in KD adipocytes, suggesting reduced infection resistance in KD. Further studies revealed that the interferon-activated interferon signaling pathway was significantly inhibited by iron but activated considerably by H_2_O_2_. This indicates excessive ROS exhaustion and iron overload in KD adipocytes contribute to impaired interferon signaling. Although it was shown that GPX4 was able to activate the interferon signaling pathway^34^, interferon was significantly reduced rather than enhanced in KD adipocytes with high GPX4 expression. This seemingly paradoxical phenomenon may be related to excessive ROS depletion and the predominant role of iron overload in KD adipocytes. Adipocytes also can secrete interferons, especially IFN-α and IFN-γ, and interferons can modulate immunity and control collagen and extracellular matrix synthesis 35. Thus, the downregulated interferon signaling we found in KD adipocytes may be an essential prerequisite and basis for their development of AMT. In addition, inflammatory activation and immune dysregulation in KD may also be associated with reduced adipocyte interferon signaling pathways. In the future, it may be possible to consider modulating anti-infection, inflammatory activation, immune dysregulation, and collagen deposition in KD and even reducing the risk of KD development by inhibiting iron overload and ferroptosis resistance in adipocytes to revert to interferon sensitivity.

GPX4 is an important catalyzing enzyme for the synthesis of glutathione, and it is also through glutathione that GPX4 exerts its potent antioxidant capacity. In fact, glutathione synthesis requires a large amount of cystine as a metabolic substrate 36. We found that KD adipocytes had significantly elevated expression of the cystine receptor system Xc^-^ in addition to high expression of GPX4, suggesting that the cellular demand for uptake of extracellular cystine is also enhanced. As early as 2008, M Christine McGahan et al. reported that iron promotes cystine uptake in lens epithelial and retinal pigment epithelial cells, favoring glutathione synthesis 37. This suggests that GPX4-induced iron overload can positively enhance cystine uptake, further strengthening the antioxidant capacity. An important question is where the cystine uptake by adipocytes comes from. Considering the neighborhood of adipocytes and fibroblasts in the skin, we first hypothesized that fibroblasts might synthesize and secrete large amounts of cysteine. Thus, we found that the cystine synthesis gene CBS and the exocytosis gene CTNS were significantly higher in KD fibroblasts than in KN. Blocking cystine synthesis in fibroblasts effectively inhibited the promotion of ferroptosis resistance in adipocytes by fibroblasts. A recent study has shown that elevated homocysteine, a substrate for cysteine synthesis, significantly promotes skin inflammation and fibrosis, while folate exhibits a mitigating effect. In the future, focusing on the entire cystine metabolism process may be a promising intervention strategy for keloids 38. Another critical question is how adipocyte abnormalities drive KD fibrosis. In Figure 2, we have found that KD adipocytes highly express SLC40A1, indicating enhanced adipocyte iron efflux. We further found enhanced TFRC and ZIP8 expression in fibroblasts, suggesting enhanced fibroblast iron uptake. Excess iron stimulation did induce fibroblast activation as well. These results suggest that both adipocytes and fibroblasts are in an open state, which facilitates abnormal cross-talk between the two cell types via iron and cystine transport. We have discovered RSL3 as a potential inhibitor of GPX4 and DFO as an effective chelator for iron overload resulting from GPX4-induced iron deposition. These present the possibility for balanced modulation of ferroptosis and iron overload, which might substantially enhance therapeutic methods focused on avoiding and managing keloids. Considering that other cells may also benefit from the anti-fibrotic effects exerted by RSL3 and DFO, it is worth looking forward to designing adipocyte-specific means of GPX4 inhibition in the future, e.g., by targeting siRNA delivery technologies or Cre-lox genetic models, to more clearly attribute adipocyte ferroptosis resistance. Recently, Fudi Wang's group found that adipocyte-derived ferroptotic signaling is significantly downregulated in obese patients and that activation of adipocyte ferroptosis effectively reduces obesity 39, 40. This result emphasizes the essential function of ferroptosis signaling in maintaining adipocyte homeostasis, a fascinating area of study that we have identified in our research. In addition, the function and mechanism of ferroptosis resistance signaling in regulating adipocytes, together with the potential targets of RSL-3, DFO, and interferon we identified in this study, will also benefit the clinical intervention of obesity diseases.

Collectively, we report that the keloid adipocytes exhibited significant resistance to ferroptosis, iron overload, and interferon non-responsiveness. Moreover, the high expression of GPX4 was a critical factor contributing to these abnormalities in adipocytes. Here, we summarized these results: ① The overexpression of GPX4 induced iron overload in adipocytes, facilitating their transformation into fibroblasts (adipocyte-myofibroblast transition, AMT). ② The excessive depletion of reactive oxygen species (ROS) by GPX4 and iron overload jointly suppressed the interferon signaling pathway. This downregulation of interferon signaling further exacerbated the AMT phenotype in adipocytes. ③ On the one hand, adipocytes acquired collagen secretion capabilities post-AMT; on the other hand, they established a microenvironment conducive to fibrosis, thereby promoting the proliferation, activation, and collagen deposition of surrounding fibroblasts. ④ Adipocytes induced by GPX4 experienced iron overload and exhibited elevated SLC40A1 expression, delivering substantial amounts of iron ions to fibroblasts for their activation. ⑤ Fibroblasts, in turn, overexpressed CBS and CTNS to synthesize and exocytose cystine in large quantities, providing substrates with high GPX4 activity back to adipocytes. This abnormal cellular communication between adipocytes and fibroblasts occurred via the exchange of iron and cystine. ⑥ The application of the GPX4 inhibitor RSL-3 and the iron chelating agent DFO significantly alleviated keloid progression in nude mice.

## Supplementary Material

Supplementary figures and tables.
