# Supplementary material for: Ferroptosis-Resistant Adipocytes Drive Keloid Pathogenesis via GPX4-Mediated Adipocyte-Mesenchymal Transition and Iron-Cystine Metabolic Communication
Source: Int J Biol Sci. 2025 Jul 28;21(11):5097–115. doi: 10.7150/ijbs.114930 (PMC12374836; doi:10.7150/ijbs.114930)
Supplement: Supplementary file 1 — Supplementary figures and tables. [file ijbsv21p5097s1.pdf]

Supplemental Data

Supplemental Figures

Supplemental Figure 1

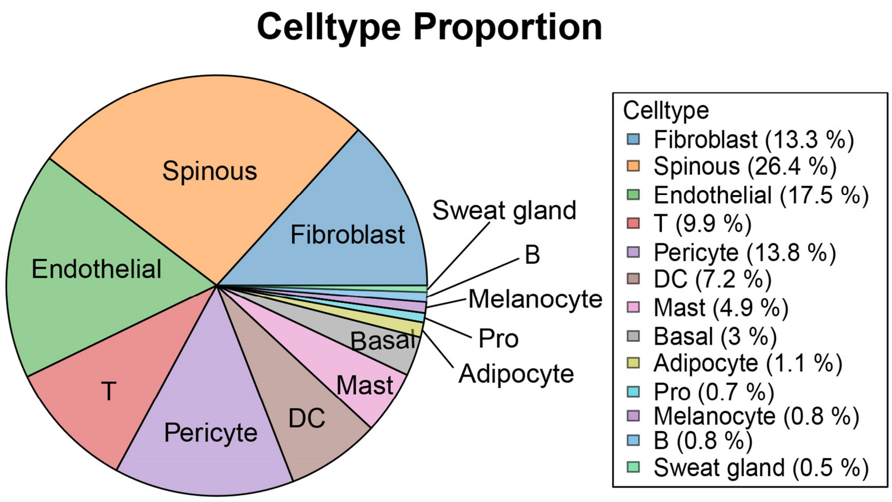

The relative abundance and distribution of major cell populations in KN and KD skins.

Supplemental Figure 2

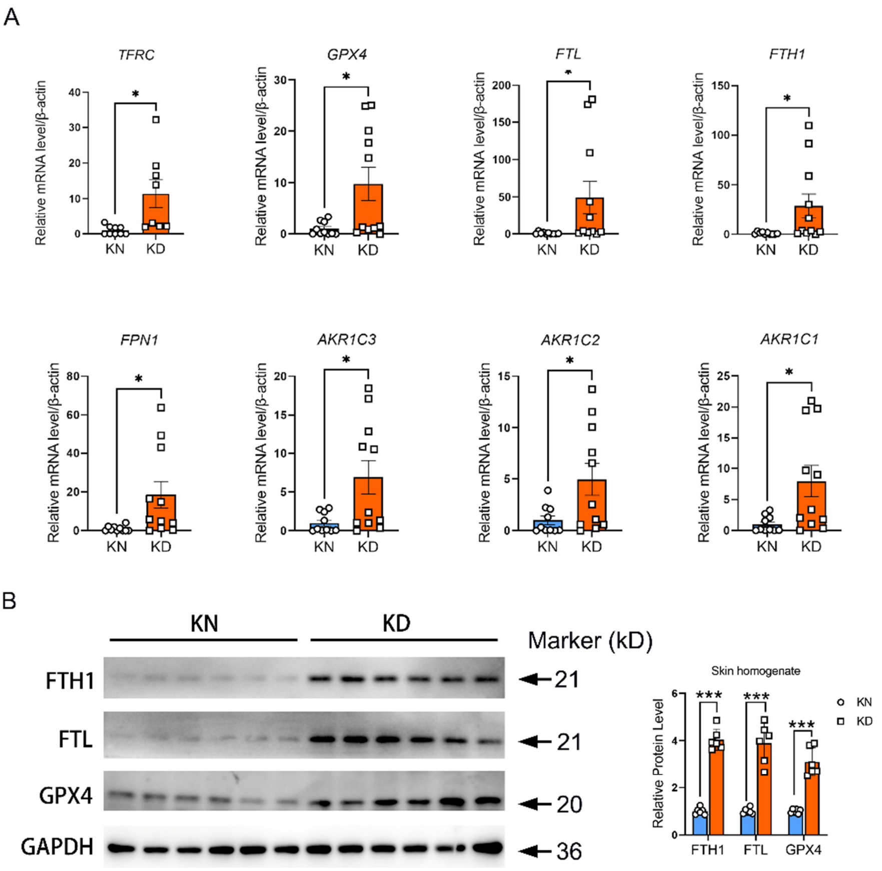

Supplementary Figure 2. Increased iron metabolism and antioxidant genes in skin tissue homogenate. (A) mRNA levels of iron metabolism and antioxidant genes in skin tissue homogenate. N ≥ 10 per group. **(B)** FTH1, FTL, and GPX4 protein levels in skin tissue homogenate. N = 6 per group. The student's t-test calculates data in A. Data in B are evaluated using the Student's t-test and corrected using the Benjamini-Hochberg procedure. Data are represented as mean ± SEM. *P < 0.05; **P < 0.001; ***P < 0.001.

Supplemental Figure 3

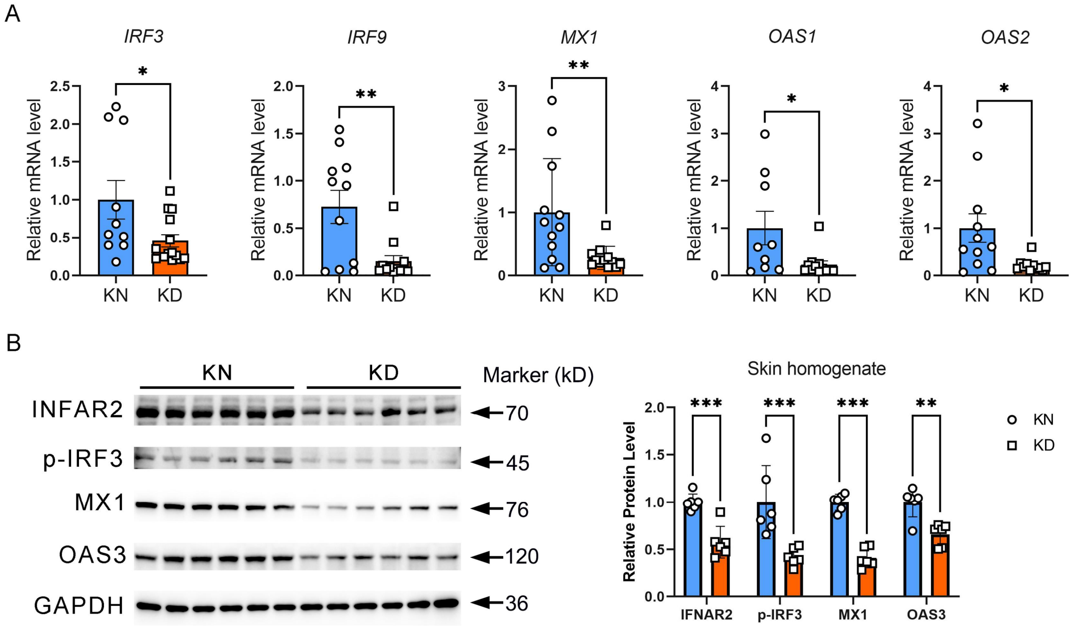

Supplementary Figure 3. Decreased interferon signaling in skin tissue homogenate. (A) mRNA levels of *IRF3, IRF9, MX1, OAS1*, and *OAS2* genes in skin tissue homogenate. N ≥ 10 per group. **(B)** IFNAR2, p-IRF3, MX1, and OAS3 protein levels in skin tissue homogenate. N = 6 per group. The student's t-test calculates all the data in A. Data are represented as mean ± SEM. *P < 0.05; **P < 0.001; ***P < 0.001.

Supplemental Tables

Supplemental Table S1 Gene panel for evaluating ferroptosis resistance scores.

Gene Symbol	Official Full Name (NCBI)
SLC7A11	solute carrier family 7 member 11
GPX4	glutathione peroxidase 4
AKR1C1	aldo-keto reductase family 1 member C1
AKR1C2	aldo-keto reductase family 1 member C2
AKR1C3	aldo-keto reductase family 1 member C3
RB1	RB transcriptional corepressor 1
HSPB1	heat shock protein family B (small) member 1
HSF1	heat shock transcription factor 1
GCLC	glutamate-cysteine ligase catalytic subunit
NFE2L2	nuclear factor, erythroid 2 like 2
SQSTM1	sequestosome 1
NQO1	NAD(P)H quinone dehydrogenase 1
HMOX1	heme oxygenase 1
FTH1	ferritin heavy chain 1
MUC1	mucin 1, cell surface associated
SLC3A2	solute carrier family 3 member 2
MT1G	metallothionein 1G
SLC40A1	solute carrier family 40 member 1
CISD1	CDGSH iron sulfur domain 1
FANCD2	FA complementation group D2
FTMT	ferritin mitochondrial
HSPA5	heat shock protein family A (Hsp70) member 5
ATF4	activating transcription factor 4
TP53	tumor protein p53
HELLS	helicase, lymphoid-specific
SCD	stearoyl-CoA desaturase
FADS2	fatty acid desaturase 2
SRC	SRC proto-oncogene, non-receptor tyrosine kinase
STAT3	signal transducer and activator of transcription 3
PML	promyelocytic leukemia
MTOR	mechanistic target of rapamycin kinase
NFS1	NFS1 cysteine desulfurase
TP63	tumor protein p63
CDKN1A	cyclin dependent kinase inhibitor 1A
ENPP2	ectonucleotide pyrophosphatase/phosphodiesterase 2
VDAC2	voltage dependent anion channel 2
FH	fumarate hydratase
CISD2	CDGSH iron sulfur domain 2

CBS	cystathionine beta-synthase
ISCU	iron-sulfur cluster assembly enzyme
ACSL3	acyl-CoA synthetase long chain family member 3
OTUB1	OTU deubiquitinase, ubiquitin aldehyde binding 1
CD44	CD44 molecule
BRD4	bromodomain containing 4
PRDX6	peroxiredoxin 6
SESN2	sestrin 2
NF2	NF2, moesin-ezrin-radixin like (MERLIN) tumor suppressor
ARNTL	aryl hydrocarbon receptor nuclear translocator like
HIF1A	hypoxia inducible factor 1 subunit alpha
JUN	Jun proto-oncogene, AP-1 transcription factor subunit
CA9	carbonic anhydrase 9
TMBIM4	transmembrane BAX inhibitor motif containing 4
PLIN2	perilipin 2
AIFM2	apoptosis inducing factor mitochondria associated 2
LAMP2	lysosomal associated membrane protein 2
ZFP36	ZFP36 ring finger protein
PROM2	prominin 2
CHMP5	charged multivesicular body protein 5
CHMP6	charged multivesicular body protein 6
CAV1	caveolin 1
GCH1	GTP cyclohydrolase 1
SIRT3	sirtuin 3
DAZAP1	DAZ associated protein 1
PIR	pirin
FTL	ferritin light chain
HCAR1	hydroxycarboxylic acid receptor 1
SLC16A1	solute carrier family 16 member 1
RRM2	ribonucleotide reductase regulatory subunit M2
NR4A1	nuclear receptor subfamily 4 group A member 1
PIK3CA	phosphatidylinositol-4,5-bisphosphate 3-kinase catalytic subunit alpha
RPTOR	regulatory associated protein of MTOR complex 1
SREBF1	sterol regulatory element binding transcription factor 1
SREBF2	sterol regulatory element binding transcription factor 2
FZD7	frizzled class receptor 7
P4HB	prolyl 4-hydroxylase subunit beta
NT5DC2	5'-nucleotidase domain containing 2
BCAT2	branched chain amino acid transaminase 2
PLA2G6	phospholipase A2 group VI
PARK7	Parkinsonism associated deglycase
FXN	frataxin

SUV39H1	suppressor of variegation 3-9 homolog 1
ATF2	activating transcription factor 2
ACOT1	acyl-CoA thioesterase 1
ALDH3A2	aldehyde dehydrogenase 3 family member A2
STK11	serine/threonine kinase 11
FNDC5	fibronectin type III domain containing 5
CDH1	cadherin 1
NEDD4L	NEDD4 like E3 ubiquitin protein ligase
TF	transferrin
BRD2	bromodomain containing 2
BRD3	bromodomain containing 3
BRDT	bromodomain testis associated
DECR1	2,4-dienoyl-CoA reductase 1
GLRX5	glutaredoxin 5
NCOA3	nuclear receptor coactivator 3
NR5A2	nuclear receptor subfamily 5 group A member 2
PANX2	pannexin 2
TFAP2A	transcription factor AP-2 alpha
CP	ceruloplasmin
ARF6	ADP ribosylation factor 6
GDF15	growth differentiation factor 15
ABHD12	abhydrolase domain containing 12
PPP1R13L	protein phosphatase 1 regulatory subunit 13 like
TFAM	transcription factor A, mitochondrial
KDM3B	lysine demethylase 3B
AHCY	adenosylhomocysteinase
IDH2	isocitrate dehydrogenase (NADP(+)) 2
PPARA	peroxisome proliferator activated receptor alpha
NOS2	nitric oxide synthase 2
SIAH2	siah E3 ubiquitin protein ligase 2
RELA	RELA proto-oncogene, NF-kB subunit
PRKAA2	protein kinase AMP-activated catalytic subunit alpha 2
VDR	vitamin D receptor
NEDD4	NEDD4 E3 ubiquitin protein ligase
PRDX1	peroxiredoxin 1
AR	androgen receptor
MTF1	metal regulatory transcription factor 1
COPZ1	COPI coat complex subunit zeta 1
NUPR1	nuclear protein 1, transcriptional regulator
USP35	ubiquitin specific peptidase 35
NEAT1	nuclear paraspeckle assembly transcript 1
PARP1	poly(ADP-ribose) polymerase 1
PARP2	poly(ADP-ribose) polymerase 2

PARP3	poly(ADP-ribose) polymerase 3
PARP4	poly(ADP-ribose) polymerase family member 4
PARP6	poly(ADP-ribose) polymerase family member 6
PARP8	poly(ADP-ribose) polymerase family member 8
PARP9	poly (ADP-ribose) polymerase family member 9
PARP10	poly(ADP-ribose) polymerase family member 10
PARP11	poly(ADP-ribose) polymerase family member 11
PARP12	poly(ADP-ribose) polymerase family member 12
PARP14	poly(ADP-ribose) polymerase family member 14
PARP15	poly(ADP-ribose) polymerase family member 15
PARP16	poly(ADP-ribose) polymerase family member 16
PDSS2	decaprenyl diphosphate synthase subunit 2
TXN	thioredoxin
SENP1	SUMO specific peptidase 1
OIP5-AS1	OIP5 antisense RNA 1
CREB1	cAMP responsive element binding protein 1
CREB3	cAMP responsive element binding protein 3
CREB5	cAMP responsive element binding protein 5
GOT1	glutamic-oxaloacetic transaminase 1
TFRC	transferrin receptor
BEX1	brain expressed X-linked 1
ASAH2	N-acylsphingosine amidohydrolase 2
FABP4	fatty acid binding protein 4
AKT1S1	AKT1 substrate 1
MLST8	MTOR associated protein, LST8 homolog
SIRT1	sirtuin 1
TYRO3	TYRO3 protein tyrosine kinase
SIRT6	sirtuin 6
TMSB4X	thymosin beta 4, X-linked
TMSB4Y	thymosin beta 4, Y-linked
KIF20A	kinesin family member 20A
ECH1	enoyl-CoA hydratase 1
ETV4	ETS variant transcription factor 4
MEG8	maternally expressed 8
VCP	valosin containing protein
RBMS1	RNA binding motif single stranded interacting protein 1
KDM4A	lysine demethylase 4A
MGST1	microsomal glutathione S-transferase 1
MPC1	mitochondrial pyruvate carrier 1
CHMP1A	charged multivesicular body protein 1A
CAMKK2	calcium/calmodulin dependent protein kinase kinase 2
SOX2	SRY-box transcription factor 2
SRSF9	serine and arginine rich splicing factor 9

PROK2	prokineticin 2
SIRT2	sirtuin 2
MEF2C	myocyte enhancer factor 2C
EZH2	enhancer of zeste 2 polycomb repressive complex 2 subunit
SMPD1	sphingomyelin phosphodiesterase 1
ADAMTS13	ADAM metallopeptidase with thrombospondin type 1 motif 13
CDC25A	cell division cycle 25A
G6PD	glucose-6-phosphate dehydrogenase
PPARD	peroxisome proliferator activated receptor delta
ENO3	enolase 3
LCN2	lipocalin 2
TRIB2	tribbles pseudokinase 2
DHODH	dihydroorotate dehydrogenase
PDK4	pyruvate dehydrogenase kinase 4
MIR9-3HG	MIR9-3 host gene
IL6	interleukin 6
PTPN18	protein tyrosine phosphatase non-receptor type 18
ABCC5	ATP binding cassette subfamily C member 5
CISD3	CDGSH iron sulfur domain 3
MS4A15	membrane spanning 4-domains A15
FURIN	furin, paired basic amino acid cleaving enzyme
GALNT14	polypeptide N-acetylgalactosaminyltransferase 14
KLHDC3	kelch domain containing 3
MAPKAP1	MAPK associated protein 1
PRR5	proline rich 5
RICTOR	RPTOR independent companion of MTOR complex 2
GSTM1	glutathione S-transferase mu 1
RARRES2	retinoic acid receptor responder 2
USP11	ubiquitin specific peptidase 11

Supplemental Table S2

**Characteristics of paired keloid patients (KD and KN)**	
**Age (years), mean ± SD**	35±9
**Gender**	
Female	N=7
Male	N=5
**Site of Specimen**	
Ear	N=3
Chest	N=6
Shoulder	N=3

**Characteristics of unpaired keloid patients (KD)**	
**Age (years), mean ± SD**	40 ± 4
**Gender**	
Female	N=10
Male	N=8
**Site of Specimen**	
Chest	N=5
Ear	N=3
Hip	N=3
Shoulder	N=5
Neck	N=2

N, Number of patients

Supplemental Table S3

qRT-PCR Primers	Sequence (5’-3’)
α-SMA	F: CTGAACGAGAACCAAGTGCG;<br>R: ACGAACCTCTTGCACATTTGA
ACSL4	F: ACTGGCCGACCTAAGGGAG;<br>R: GCCAAAGGCAAGTAGCCAATA
ADIPOQ	F: TGCTGGGAGCTGTTCTACTG;<br>R: TACTCCGGTTTCACCGATGTC
AKR1C1	F: TTCATGCCTGTCCTGGGATTT;<br>R: CTGGCTTTACAGACACTGGAAAA
AKR1C2	F: CAACTTCAACCACAGGCTGC;<br>R: GTCTGATGCGCTGCTCATTG
AKR1C3	F: GAGAGCAGAAGACCGAAAGGA;<br>R: CACAACACCACGTTATCGGG
COL1A1	F: GTGGGCAACGACTCTGGAC;<br>R: GCTTCGACATCAGCATTCCTCA
COL1A2	F: GATCACCCGAATGGCTATGAAT;<br>R: GGGGTCACAGTTGTCAATGTT
COL3A1	F: CACAACACGCTGTTCGGCTA;<br>R: CGATCCTGCATCTGTAAATCGC
EIF2AK2	F: GCCGCTAAACTTGCATATCTTCA;<br>R: TCACACGTAGTAGCAAAAGAACC
FN1	F: CGGTGGCTGTCAGTCAAAG;<br>R: AAACCTCGGCTTCCTCCATAA
FTH1	F: CCCCCATTTGTGTGACTTCAT;<br>R: GCCCGAGGCTTAGCTTTCATT
FTL	F: CAGCCTGGTCAATTTGTACCT;<br>R: GCCAATTCGCGGAAGAAGTG
GAPDH	F: GGAGCGAGATCCCTCCAAAAT;<br>R: GGCTGTTGTCATACTTCTCATGG
GPX4	F: GAGGCAAGACCGAAGTAAACTAC;<br>R: CCGAACTGGTTACACGGGAA
IRF3	F: AGAGGCTCGTGATGGTCAAG;<br>R: AGGTCCACAGTATTCTCCAGG
IRF9	F: GCCCTACAAGGTGTATCAGTTG;<br>R: TGCTGTCGCTTTGATGGTACT
ISG15	F: CGCAGATCACCCAGAAGATCG;<br>R: TTCGTCGCATTTGTCCACCA
MX1	F: GTTTCCGAAGTGGACATCGCA;<br>R: CTGCACAGGTTGTTCTCAGC
MX2	F: CAGAGGCAGCGGAATCGTAA;<br>R: TGAAGCTCTAGCTCGGTGTTC

NFE2L2	F: TCAGCGACGGAAAGAGTATGA;<br>R: CCACTGGTTTCTGACTGGATGT
OAS1	F: TGTCCAAGGTGGTAAAGGGTG;<br>R: CCGGCGATTTAACTGATCCTG
OAS2	F: CTCAGAAGCTGGGTTGGTTTAT;<br>R: ACCATCTCGTCGATCAGTGTC
PLIN1	F: TGTGCAATGCCTATGAGAAGG;<br>R: AGGGCGGGGATCTTTTCCT
SLC3A2	F: TGAATGAGTTAGAGCCCGAGA;<br>R: GTCTTCCGCCACCTTGATCTT
SLC40A1	F: CTACTTGGGGAGATCGGATGT;<br>R: CTGGGCCACTTTAAGTCTAGC
SLC7A11	F: TCTCCAAAGGAGGTTACCTGC;<br>R: AGACTCCCCTCAGTAAAGTGAC
TFRC	F: ACCATTGTCATATACCCGGTTCA;<br>R: CAATAGCCCAAGTAGCCAATCAT
TGF-β1	F: GGCCAGATCCTGTCCAAGC;<br>R: GTGGGTTTCCACCATTAGCAC
